# Tumor-associated macrophages confer colorectal cancer 5-fluorouracil resistance by promoting MRP1 membrane translocation via an intercellular CXCL17/CXCL22–CCR4–ATF6–GRP78 axis

**DOI:** 10.1038/s41419-023-06108-0

**Published:** 2023-09-01

**Authors:** Lichao Zhang, Xiaoqing Lu, Yuanzhi Xu, Xiaoqin La, Jinmiao Tian, Aiping Li, Hanqing Li, Changxin Wu, Yanfeng Xi, Guisheng Song, Zhaocai Zhou, Wenqi Bai, Liwei An, Zhuoyu Li

**Affiliations:** 1 Institutes of Biomedical Sciences, Shanxi University, 030006 Taiyuan, China; 2 Shanxi Province Cancer Hospital, Shanxi Hospital Affiliated to Cancer Hospital of Chinese Academy of Medical Sciences, Cancer Hospital Affiliated to Shanxi Medical University, Taiyuan, China; 3 Department of Stomatology, Shanghai Tenth People’s Hospital, Department of Biochemistry and Molecular Biology, Tongji University School of Medicine, 200072 Shanghai, China; 4 Institute of Biotechnology, Shanxi University, 030006 Taiyuan, China; 5 Modern Research Center for traditional Chinese medicine, Shanxi University, 030006 Taiyuan, China; 6 School of Life Science, Shanxi University, 030006 Taiyuan, China; 7 Department of Medicine, University of Minnesota, Minneapolis, MN 55455 USA; 8 State Key Laboratory of Genetic Engineering, Zhongshan Hospital, School of Life Sciences, Fudan University, 200438 Shanghai, China

**Keywords:** Colon cancer, Cancer microenvironment, Cancer therapeutic resistance

## Abstract

Chemotherapy represents a major type of clinical treatment against colorectal cancer (CRC). Aberrant drug efflux mediated by transporters acts as a key approach for tumor cells to acquire chemotherapy resistance. Increasing evidence implies that tumor-associated macrophages (TAMs) play a pivotal role in both tumorigenesis and drug resistance. Nevertheless, the specific mechanism through which TAMs regulate drug efflux remains elusive. Here, we discovered that TAMs endow CRC cells with resistance to 5-fluorouracil (5-FU) treatment via a cell-cell interaction-mediated MRP1-dependent drug efflux process. Mechanistically, TAM-secreted C-C motif chemokine ligand 17 (CCL17) and CCL22, via membrane receptor CCR4, activated the PI3K/AKT pathway in CRC tumor cells. Specifically, phosphorylation of AKT inactivated IP3R and induced calcium aggregation in the ER, resulting in the activation of ATF6 and upregulation of GRP78. Accordingly, excessive GRP78 can interact with MRP1 and promote its translocation to the cell membrane, causing TAM-induced 5-FU efflux. Taken together, our results demonstrated that TAMs promote CRC chemotherapy resistance via elevating the expression of GRP78 to promote the membrane translocation of MRP1 and drug efflux, providing direct proof for TAM-induced drug resistance.

## Introduction

Colorectal cancer (CRC) ranks as the third most common and lethal tumor type worldwide [1]. Currently, surgery combined with radio-chemotherapy represents the two major treatments for CRC. Of note, chemotherapy can be applied at different stages of CRC treatment, and 5-Fluorouracil (5-FU) is the most frequently used chemical drug for CRC chemotherapy [2]. However, drug resistance issues can lead to a decrease in the efficiency of chemotherapy or even failure, representing a threat to 5-FU widespread in clinical application [3]. The resistance can be intrinsic (de novo) or acquired (therapy-induced), which could be induced by aberrant gene expression, gene mutations, angiogenesis, deregulation of autophagy, epithelial–mesenchymal transition (EMT), and the presence of stem cells [4, 5]. Among them, the most common reason for acquired drug resistance in cancer cells is the expression and membrane translocation of energy-dependent transporters which detect and eject anticancer drugs from cells [6]. P-glycoprotein (P-gp), also known as multidrug transporter (MDR), is widely expressed in diverse cancers and participates not only in drug efflux but also in the transport of nutrients and other biologically important molecules across plasma membranes [7]. On the other hand, multidrug resistance-associated protein (MRP) is another ABC family member. MRP mainly transports negatively charged antitumor and antiviral compounds (e.g. 5-FU) and has been shown to promote the drug resistance of cancer cells [8]. In addition, many other transporters (breast cancer resistance protein, BCRP; and lung resistance-related protein, LRP) were found in specific tissue first, however, further research revealed that they can also locate in many other tumor tissues [9, 10].

The heterogeneity of the tumor microenvironment (TME) acts as the key factor affecting the efficiency of tumor chemotherapy [11]. TME is a complex physical and biochemical ecosystem, composed of diverse cell types including cancer cells, stromal tissues, immune cells, and extracellular matrix [12]. Among them, macrophages are the largest population of immune cells in the TME [13], and are closely related to tumor development [14]. Based on their functions, macrophages are classified into classically activated M1 phenotype and alternatively activated M2 phenotype. M1 macrophages have strong microbicidal and tumoricidal activity, while M2-polarized macrophages have increased tissue repair and promote angiogenesis abilities [15]. Specifically, tumor-associated macrophages (TAMs) are most likely M2-polarized macrophages in phenotype and function, and the infiltration of TAMs is closely related to the poor prognosis of tumor patients [16]. Previous studies on the regulatory role of TAMs on drug resistance mainly focused on promoting survival and anti-apoptosis [17, 18]. However, as the main participants of acquired drug resistance, the role of TAMs in regulating drug transports and subsequent tumor chemotherapy resistance is still unclear.

Glucose-regulated protein 78 (GRP78) is a chaperone of the endoplasmic reticulum (ER), and mainly facilitates proper protein folding and assists misfolded proteins to be degraded via the proteasome pathway [19]. Due to aberrant proliferation properties, the tumor microenvironment undergoes contexts such as hypoxia, acidosis, and glucose deprivation, resulting in perturbation of ER homeostasis and activated expression of GRP78. Excessive GRP78 is involved in almost all aspects of the malignant transformation of tumors [20–22]. Specifically, elevated expression of GRP78 is positively correlated with chemotherapy resistance [20], nevertheless, the inter-relationship of GRP78 overexpression and drug transporters needs to be further investigated.

Here in this work, we first revealed that GRP78 was an important effector of TAMs-induced 5-FU resistance in CRC. Mechanistically, GRP78 may promote chemotherapy resistance via the multidrug resistance-associated protein MRP1. These results indicated that TAMs were able to directly regulate the efflux of anticancer drugs from cells, and GRP78 was an important intermediate between TAMs and MRP1-mediated tumor drug resistance. Therefore, we hypothesized that TAMs, GRP78, and drug transporters synthetically modulate tumor chemotherapy resistance. Here, transcriptomics, molecular and cellular approaches, and mouse models were used to test our hypothesis and determine how TAMs regulate MRP1 by activating the expression of GRP78.

## Materials and methods

### Cell culture, macrophages differentiation, and flow cytometric analysis

Human colon cancer cell lines DLD1, SW480, and SW620 were cultured in RPMI-1640 medium which was containing 10% fetal bovine serum (FBS). Human monocytes THP-1 cells were cultured in RPMI-1640 medium which was containing 10% FBS and supplemented with 50 pM β-mercaptoethanol.

The human peripheral blood monocytes isolation kit (Solarbio, China) was applied to isolate the human peripheral blood monocytes. The differentiations of THP-1 and hPBMC were performed as previously described [23]. Briefly, the polarization of M1 macrophages was induced by IFN-γ (20 ng/mL) and LPS (100 pg/mL); the polarization of TAMs was induced by IL-13 (20 ng/mL) and IL-4 (20 ng/mL). After incubated with the above inducing factor for 48 h, the culture supernatant was replaced by the fresh medium, and the cells were cultured for another 72 h, then the culture supernatant was collected as M1 macrophages and TAMs condition medium (M1-CM-T, TAMs-CM-T, M1-CM-H, and TAMs-CM-H), respectively.

### Transfection and siRNA knock down

Human GRP78 cDNA, different truncated mutants, and T453D mutant of GRP78 were constructed into the pLVX-AcGFP1-N1 vector. Human *GRP78* shRNAs were constructed in pLKO.1-Puro vector. pLVX-AcGFP1-N1-GRP78 or pLKO.1-Puro-shGRP78 plasmid was co-transfected into 293 T cells with psPAX2, and pMD2.G using the Calcium Phosphate method at 15:10:5 g (for a 10-cm dish). After transfection 48 h, supernatant media containing the virus was collected and concentrated with 100 kDa ultrafiltration membranes (Millipore). Concentrated viruses were used to infect DLD1 and SW480 cells in the presence of polybrene (8 μg/mL) for 48 h. To get stable cell lines, the virus-infected cell needs to be further selected with 5 μg/mL puromycin for 72 h. The Human *ATF-6* and *MRP1* siRNA was designed and synthesized by GenePharma (Shanghai, CHN), and transfected in cells by Lipofectamine 3000 (Thermo Fisher Scientific). The sequences of shRNA and siRNA are listed in Supplemental Table 3.

### MTT assay

Tumor cells were pre-incubated with macrophages condition medium (CM) 1 h. After that, 5-FU was added to the medium and incubated for another 24 h. The viability of cells was assessed by MTT assay as described previously [24].

### Colony-formation assay

DLD1 and SW620 cells were pre-incubated with CM. 1 h later, 0, 10, 20, or 40 μM 5-FU were added to the medium and incubated for 7 days. The cell colonies were fixed with paraformaldehyde and stained with crystal violet, and photographed with the stereomicroscope. After cell lysis with 1% SDS, cell lysate was tested at 570 nm.

### Co-immunoprecipitation and Western blot assays

Both Western blot and co-immunoprecipitation were performed as described previously [25]. For whole-cell extracts, cells were lysed with 20 mM Tris·HCl, pH 8.0, 100 mM NaCl, 0.5% NP-40, and 1 mM EDTA (NETN buffer) supplemented with Micrococcal Nuclease (1:5000, M0247S, NEB) on ice for 15 min. Cell lysates were boiled with 5× SDS loading buffer, separated by SDS-PAGE, transferred to polyvinylidene fluoride membranes, and immunoblotted with indicated antibodies. For co-IP experiments, cells were lysed with NETN buffer for 15 min on ice, followed by centrifugation at 12,000 rpm for 10 min at 4 °C. Supernatants were then transferred into new Eppendorf tubes and incubated with either Flag beads (L00425, GenScript) for 4 h or Protein A/G plus agarose beads (sc-2003, Santa Cruz) conjugated with indicated antibodies overnight at 4 °C with rotation. Beads were subsequently washed three times with NETN buffer and boiled with 1× SDS loading buffer. To avoid the noise of light/heavy chains, the secondary antibodies used for the immunoprecipitation assays including mouse anti-rabbit IgG LCS (A25022, 1:5000) and goat anti-mouse IgG LCS (A25012, 1:5000) were obtained from Abbkine.

### Transcriptome analysis

DLD1 cells were incubated with 20% M2-CM-H for 24 h, and the cells were collected and quickly frozen in liquid nitrogen. Subsequent transcriptomics analysis was completed by Novogene (Beijing, China).

### Differential expression analysis

The DESeq2 R package (1.20.0) was applied to the differential expression analysis of the Cont and TAMs-CM treatment groups. The genes found by DESeq2 and with an adjusted *p* value < 0.05 were assigned as differentially expressed.

### GO and KEGG enrichment analysis of differentially expressed genes

The Cluster Profiler R package was applied to Gene Ontology (GO) enrichment and KEGG pathways analysis of differentially expressed genes, GO terms with corrected *p* value < 0.05 were considered significantly enriched by differentially expressed genes.

### Pathway-gene network construction and analysis

To reveal the internal relationships among the selected DEGs and their pathways, we constructed networks and performed network analysis. We constructed a network that mapped the DEGs to their pathways. In detail, if a DEG was involved in pathways, then we connected the DEG with each of its pathway terms. Finally, the pathway-gene network was visualized using Cytoscape software v 3.8.2.

### RNA extraction and qRT-PCR analysis

The extraction of total RNA, reverse transcription of cDNA, and qRT-PCR were implemented as previously described [25]. The primers involved in this study were listed in Supplemental Table 1.

### Immunofluorescence, calcium, and ATP detection

Cells were seeded onto glass slides and suffered related treatment, and the following immunofluorescence analysis was performed as previously described [25].

For calcium detection, after fixation, slides were incubated with Fluo-3 AM and ER-tracker at 37 °C for 30 min, and the nucleus was marked with DAPI at 37 °C for 10 min. After three washes, slides were mounted and analyzed by fluorescence microscope. For the membrane-translocation detection of MRP1 and GRP78, slides were incubated with primary antibodies at 4 °C overnight and incubated with second antibodies at 25 °C for 2 h. After three washes, slides were mounted and analyzed by fluorescence microscope. The total intracellular calcium concentration was detected using the Calcium Colorimetric Assay Kit from Sigma (St. Louis, USA). The total intracellular ATP concentration was detected using the ATP Assay Kit from Sigma (St. Louis, USA).

### HPLC analysis

Briefly, after proper treatment, the cells were collected and lysed by 15 cycles of freezing and thawing, and the resultant supernatants were centrifuged (13,000 rpm, 4°C, 10 min), dried in nitrogen flow, and redissolved into mobile phase subject to 5 mL.

5-FU was accurately weighed and dissolved in methanol-water (10/90, v/v) to prepare the stock solution at the concentration of 0.904 µg/mL, and the stock solution was serially diluted with methanol–water (10/90, v/v) to obtain working solutions (six different concentrations in the range of 0.028–0.904 µg/mL). 20 μL of the above solutions were injected for analysis, and peak areas were recorded to manufacture a standard curve.

All chromatographic measurements were performed on the Waters 2695 system equipped with Empower chromatography workstation and Vensil MP C18 (4.6 mm × 250 mm, 5 μm, Tianjin Bonna-Agela Technologies Ltd.). The mobile phase consisted of (A) 90% water and (B) 10% methanol under isocratic elution for 10 min. The column flow rate was maintained at 1 mL/min, the column temperature was 25 °C, and the detection wavelength was 265 nm.

### In vivo experiment in mice

For xenograft tumor assay, 40 4-week-old male immunocompromised BALB/c mice were divided into four groups randomly (*n* = 10). In groups I and II, each mouse was injected with 2 × 10^6^ DLD1 cells subcutaneously. In group III, each mouse was injected with 1.6 × 10^6^ DLD1 cells (sh-Con) and 0.4 × 10^6^ TAMs derived from THP-1 subcutaneously. In group IV, each mouse was injected with 1.6 × 10^6^ DLD1 cells (sh-GRP78) and 0.4 × 10^6^ TAMs derived from THP-1 subcutaneously. A week later, the mouse of groups II, III, and IV were intraperitoneally injected with 5-FU at a dose of 20 mg/kg every 2 days, and the mouse of group I were injected with normal saline. The long diameter (*a*) and short diameter (*b*) were measured weekly, and the volume of the tumor was calculated by *V* = *a* × *b*^2^/2. Five weeks later, the mice were euthanized.

For AOM/DSS-induced colonic tumorigenesis model, DSS was purchased from MP Biomedicals (California, USA), and AOM was purchased from Sigma (St. Louis, USA). The model was established as the previous study. On the first day, AOM (10 mg/Kg) was injected into the peritoneal of C57BL/6 mice. After 1 week, the drinking water was replaced by a 2% DSS aqueous solution and maintained for 1 week, and then it was removed for 2 weeks intervals. The induction process of DSS was repeated 4 times to establish this model. After 11 weeks, 5-FU (20 mg/kg) or 5-FU (20 mg/kg) plus HA15 (35 mg/kg) was intraperitoneally injected every three days. After 16 weeks, the mice were sacrificed. The tumor numbers per colon were counted as previously described [26]. Tumor size was calculated as length × width (mm^2^) [26], and the tumor diameter was measured with the sliding caliper. All animal experiments were carried out by following a protocol approved by the Institutional Research Ethics Committee of Shanxi University.

### Clinical sample collection and analysis

Human samples of pre-chemotherapy colorectal tumor (*n* = 31) and relapsed colorectal tumor (therapied with FOLFOX4 chemotherapy scheme, *n* = 28) were collected from the Shanxi Cancer Hospital. Informed consent was obtained from all patients, and the tumor specimens of colon cancer patients were conducted with permission from the Institutional Research Ethics Committee of Shanxi University.

The immunohistochemistry analysis of tissue specimens was entrusted to the laboratory department of Shanxi Cancer Hospital to implement. The integrated option density (IOD) of the brown areas in the immunohistochemistry analysis was calculated by Image-Pro-Plus 6.0 software, and the IOD of every section was determined by the average IOD of five random fields.

### Statistical analysis

Statistical analysis was implemented by SPSS (Windows, version 22.0). Comparison between the two groups was evaluated using Student’s *t* test, and differences among groups were assessed using one-way analysis of variance (ANOVA). The ‘Center values’ of the box plots were defined as medians. *p* < 0.05 was considered statistically significant. Data are expressed as the mean ± SD for at least three independent experiments.

## Results

### The secretome of TAMs promotes 5-FU drug resistance in CRC tumors

**Fig. 1:**
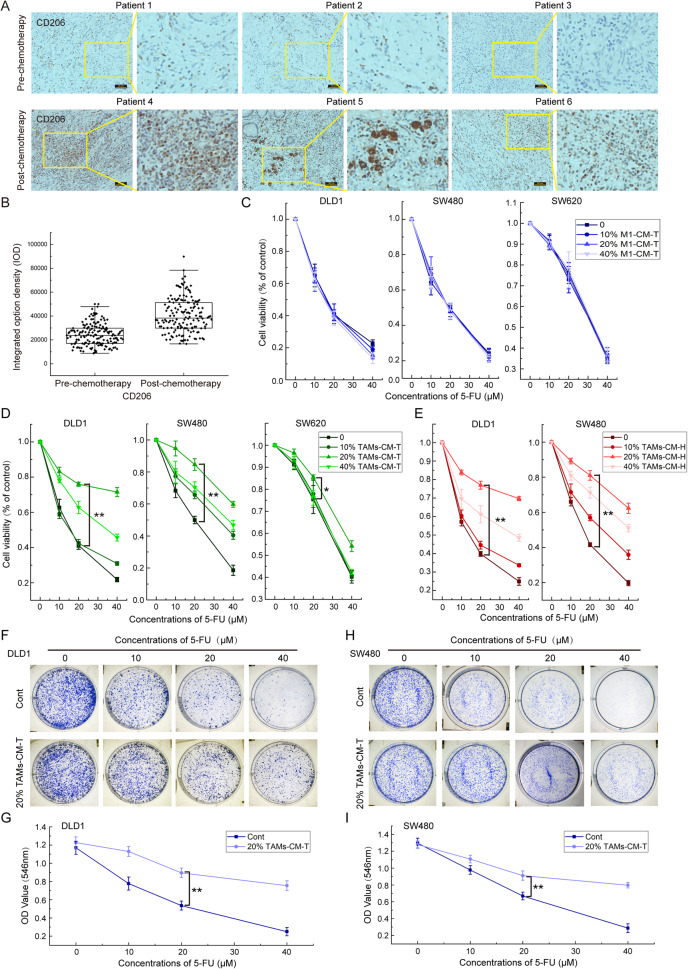
TAMs infiltration positively correlates with CRC resistance to 5-FU. **A** Immunohistochemistry was applied to detect the infiltration of TAMs (Marked with CD206) in clinical samples. **B** TAMs were marked with CD206 antibody, and the integrated option density (IOD) was used to quantify the positive areas of immunohistochemical staining. **C** Effect of M1-CM-T on the decreases of DLD1, SW480, and SW620 cell viability induced by 5-FU. **D** Effect of TAMs-CM-T on the decreases of DLD1, SW480, and SW620 cell viability induced by 5-FU; *p < 0.05, **p < 0.01. **E** Effect of TAMs-CM-H on the decreases of DLD1 and SW480 cell viability induced by 5-FU, **p < 0.01. **F** Effect of 20% TAMs-CM-T on the diminution of DLD1 cell colony formation capacity induced by 5-FU, **p < 0.01. **G** The line graph indicates the DLD1 cell colony formation capacity that was shown in panel F, **p < 0.01. **H** Effect of 20% TAMs-CM-T on the diminution of SW480 cell colony formation capacity induced by 5-FU, **p < 0.01. **I** The line graph indicated the SW480 cell colony formation capacity that was shown in **H**, **p < 0.01.

To investigate whether the infiltration of TAMs may contribute to CRC drug resistance, we collected tissues from 31 CRC patients without chemotherapy treatment and 28 cases of relapsed CRC following a standard FOLFOX4 chemotherapy scheme. IHC analysis of the CD206 signal intensity, a marker of M2-polarized macrophages, revealed that more frequency of TAMs infiltration was detected in the relapsed CRC tumors than those of untreated tumors (Fig. 1A, B). As 5-FU is the major component of the FOLFOX4 scheme for CRC clinical chemotherapy, thus we speculated that TAMs infiltration may contribute to acquired CRC drug resistance to 5-FU.

To validate this hypothesis, we first collected the conditioned medium from cultured M1 macrophages (hereafter referred to as M1-CM) and TAMs (hereafter referred to as TAMs-CM) which are individually induced from either THP-1 or hPBMC cells following a standard procedure as previously described [23] (Fig. S1), and subsequently investigated the effects of these conditioned medium on 5-FU sensitivity in CRC cancer cells. Interestingly, we did not detect any effect on cell viability of DLD1, SW480, and SW620 in response to 5-FU treatment upon co-treatment with M1-CM (Fig. 1C). Nevertheless, treatment of conditioned medium from THP-1-derived TAMs (TAMs-CM-T) could dose-dependently relieve the 5-FU sensitivity in all of the three CRC cell lines (Fig. 1D). Specifically, 20% TAMs-CM-T exhibited the best protective effect against 5-FU (Fig. 1D). Similarly, the conditioned medium from hPBMC derived TAMs (TAMs-CM-H) also robustly induced DLD1 and SW480 cells more resistant to 5-FU (Fig. 1E). Moreover, colony formation experiments were performed to further validate the ability of TAMs-CM to establish 5-FU resistance in CRC cells. As expected, the colony numbers were gradually reduced upon the increased dose of 5-FU treatment, however, co-treatment with 20% TAMs-CM-T dramatically rescued the colony formation ability under the same condition as 5-FU treatment in both DLD1 (Fig. 1F, G) and SW480 (Fig. 1H, I). Taken together, these results demonstrated that the secretome of TAMs contributes to the acquired 5-FU drug resistance in CRC tumors.

### Elevated tumoral GRP78 mainly mediated TAMs-triggered CRC 5-FU resistance

**Fig. 2:**
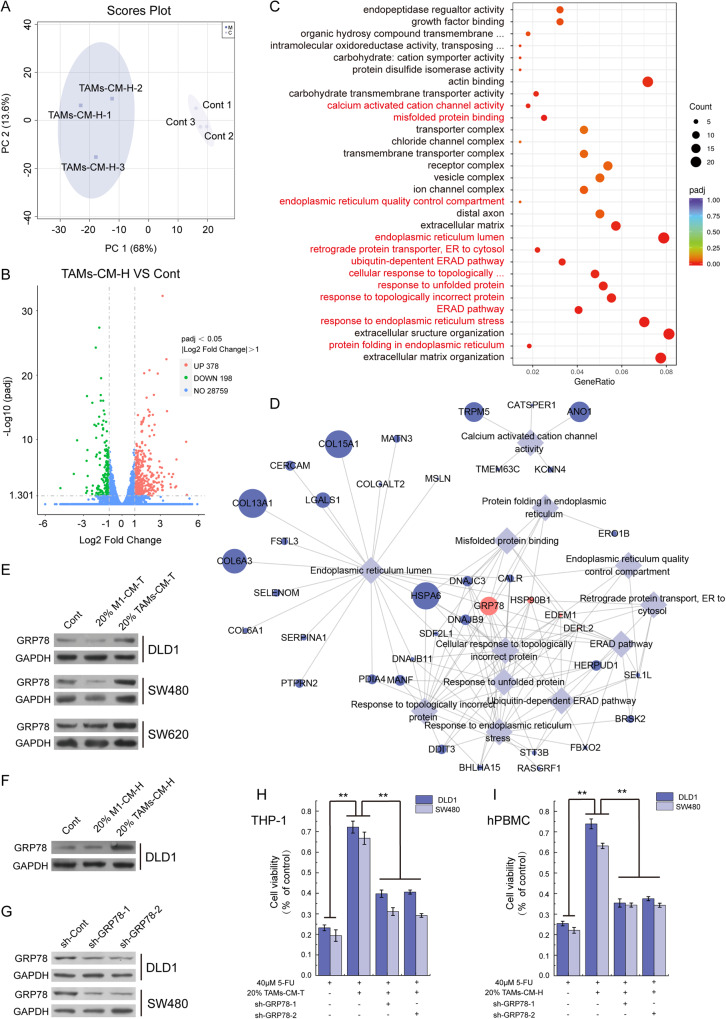
GRP78 mediated the TAMs-associated CRC resistance to 5-FU. **A** PCA score plot showing DLD1 (control) and TAMs-CM-H treated DLD1 (TAMs-CM-H) samples. **B** The volcano map illustrated the distribution of differential genes. **C** GO enrichment analysis of up-regulated genes (TAMs-CM-H VS Control). **D** The network analysis of endoplasmic reticulum-related terms and the genes involved. **E** After treatment with 20% M1-CM-T or 20% TAMs-CM-T, the level of GRP78 protein was detected by western blot in DLD1, SW480, and SW620 cells. **F** After treatment with 20% M1-CM-H or 20% TAMs-CM-H, the level of GRP78 protein was detected by western blot in DLD1 cells. **G** After the knockdown of GRP78 with shRNA in DLD1 and SW480 cells, the expression of GRP78 was detected by western blot. **H** The effect of 20% TAMs-CM-T on 5-FU resistance was detected with DLD1 and SW480 cells which with GRP78 knocking down, **p < 0.01. **I** The effect of 20% TAMs-CM-H on 5-FU resistance was detected with DLD1 and SW480 cells which with GRP78 knocking down, **p < 0.01.

To explore the underlined mechanism (s) of TAM in mediating the 5-FU resistance in CRC, we next performed transcriptome analysis to seek the alteration of signal pathways upon TAMs-CM treatment. The unsupervised PCA score plot illustrated that there were separation characteristics between the control group and TAMs-CM-H treated group, and the clustering characteristics within groups, which showed good repeatability between biological repeats and the large differences between the two groups (Fig. 2A). Next, the differential gene analysis showed that there were 378 genes upregulated, whereas 198 genes were down-regulated upon TAMs-CM treatment (Fig. 2B). Since there are twice as many up-regulated genes as down-regulated genes, we speculate that up-regulated genes may play a more important role in macrophage-mediated tumor resistance, so we performed GO enrichment analysis of the 378 upregulated genes and identified a total of 30 terms which were significantly altered by TAMs-CM treatment (Figs. 2C and S2). Intriguingly, we found that 12 of these 30 terms were related to the endoplasmic reticulum responses (marked with red, Fig. 2C). Similarly, we also performed the GO enrichment analysis of the 198 downregulated genes which mainly revealed chromatin structure terms (Fig. S3). Moreover, network analysis of these 12 terms revealed a list of genes frequently occurred in these endoplasmic reticulum-related responses (Fig. 2D and Table S2). Among them, four genes including *GRP78*, *HSP90B1*, *EDEM1*, and *DERL2* appeared in 9 out of these 12 GO processes (Table S2), and the *GRP78* mRNA level showed the highest fold change of up-regulation upon treatment (Fig. 2D and Table S2). Therefore, we speculated that GRP78 may play an important role in TAMs secretome-mediated CRC resistance to 5-FU.

Next, we examined the level of GRP78 protein in CRC cells treated with either 20% M1-CM-T or TAM-CM-T, and consistently found that the TAMs-CM-T, but not the M1-CM-T, robustly increased the GRP78 expression (Fig. 2E). Similar observation was also recapitulated in DLD1 treated with TAM-CM-H (Fig. 2F). To further examine whether GRP78 indeed mediated the 5-Fu resistant acquired by TAMs-CM, we depleted GRP78 using two shRNAs (Fig. 2G), and further assessed the ability of TAM-CM to induce 5-FU resistance in CRC cells. Indeed, though 20% TAM-CM-T significantly elevated the cell viability upon 5-FU treatment, further GRP78 ablation greatly attenuated such ability in both DLD1 and SW480 cell lines (Fig. 2H). In addition, we reproducibly observed such GRP78-dependent establishment of 5-FU resistance when using 20% TAM-CM derived from hPBMCs (Fig. 2I). Collectively, these results demonstrated endoplasmic reticulum-related GRP78 upregulation mainly orchestrated the TAMs secretome-induced 5-FU resistant in CRC.

### GRP78 upregulation correlates with 5-FU resistance in relapsed CRC tumors

**Fig. 3:**
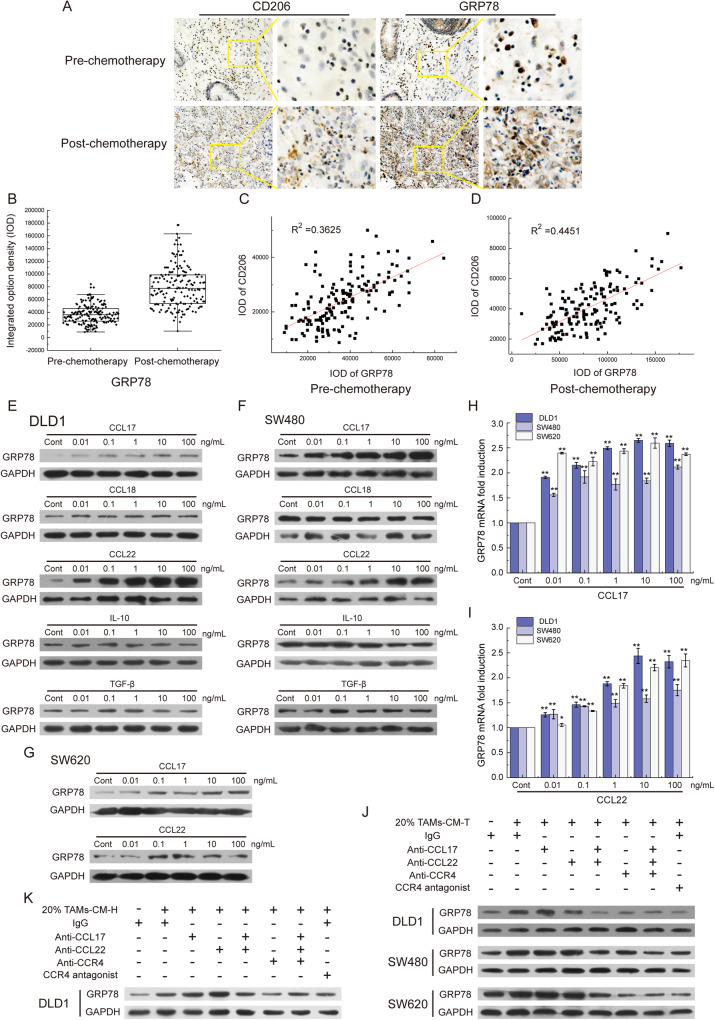
The CCL17/CCL22-CCR4 axis was responsible for GRP78 expression. **A** The level of GRP78 protein and the infiltration of TAMs (Marked with CD206) were detected in clinical samples by IHC. **B** The statistics of the integrated option density (IOD) of the brown areas in **A**. **C** The graph represented the correlation of GRP78 and CD206 in pre-chemotherapy colorectal tumors. **D** The correlation analysis of GRP78 and CD206 in post-chemotherapy colorectal tumors. **E** The level of GRP78 protein was detected in DLD1 cells treated with CCL17, CCL18, CCL22, IL-10, and TGF-β. **F** The level of GRP78 protein was detected in SW480 cells treated with CCL17, CCL18, CCL22, IL-10, and TGF-β. **G** The level of GRP78 protein was detected in SW620 cells treated with CCL17 and CCL22. **H** The level of GRP78 mRNA was detected in DLD1, SW480, and SW620 cells treated with CCL17 by qRT-PCR, **p < 0.01. **I** The level of GRP78 mRNA was detected in DLD1, SW480, and SW620 cells treated with CCL22 by qRT-PCR, *p < 0.05, **p < 0.01. **J** The level of GRP78 protein was detected in DLD1, SW480, and SW620 cells with 20% TAMs-CM-T treatment. Antibodies against CCL17, CCL22, and CCR4 and the CCR4 antagonist were applied to block the CCL17/CCL22-CCR4 pathway. **K** The level of GRP78 protein was detected in DLD1 cells with 20% TAMs-CM-H treatment. Antibodies against CCL17, CCL22, and CCR4 and the CCR4 antagonist were applied to block the CCL17/CCL22-CCR4 pathway.

Next, to validate whether GRP78 upregulation correlations with 5-FU resistance in tumor species, we examined the GRP78 expression pattern in pre-chemotherapy and relapsed CRC tumor samples via IHC staining. In resemble to the increased infiltration of TAMs (CD206 staining) in relapsed CRC tumors (Fig. 1A, B), the GRP78 signals were also dramatically upregulated in relapsed CRC tumors (Fig. 3A, B). Further linear correlation analysis revealed that infiltration of TAMs was positively correlated with GRP78 expression in pre-chemotherapy CRC tumors (*R*^2^ = 0.3625, Fig. 3C), and this correlation is much more significant in relapsed CRC tumors (*R*^2^ = 0.4451, Fig. 3D). Overall, these data indicated that tumoral GRP78 played an important role in TAMs-induced 5-FU resistance of CRC tumors.

### The CCL17/CCL22–CCR4 ligand/receptor interaction is responsible for GRP78 upregulation in CRC cells

After establishing the link between the secretome of TAMs and GRP78-dependent 5-FU resistant CRC cells, we continued to dissect the upstream regulatory network which mediates the intercellular communication between TAMs secretome and GRP78 upregulation in CRC tumor cells. Considering the major components of macrophage secretome, we speculated those cytokines may induce the expression of GRP78 in tumor cells. To test this speculation, we chose five classical TAMs-secreted cytokines including IL-10, CCL17, CCL18, CCL22, and TGF-β to examine their stimulative effects on GRP78 expression in CRC cells [16, 27]. Interestingly, this mini-screen analysis revealed that CCL17 and CCL22 treatment, but not IL-10, CCL18, or TGF-β, could dose-dependently promote GRP78 expression as analyzed by western blotting in DLD1 (Fig. 3E), SW480 cells (Fig. 3F), and SW620 cells (Fig. 3G). In line with this, the mRNA level of *GRP78* in these three cell lines was similarly upregulated in a dose-dependent manner upon either CCL17 (Fig. 3H) or CCL22 (Fig. 3I) treatment as analyzed by qPCR analysis. Moreover, ELISA analysis indeed revealed that the protein levels of CCL17 and CCL22 in 20% TAMs-CM-T were approximately 0.06 ng/mL and 0.1 ng/mL, respectively, which were much higher than in M1-CM-T (Fig. S4A, B).

After that, we pre-incubated the TAMs-CM with antibodies against CCL17 and CCL22 and further examined the stimulatory effect on GRP78 expression. As shown in Fig. 3J, we repeatedly observed the markedly increased GRP78 expression upon 20% TAMs-CM-T treatment, however, such effects were greatly attenuated in TAMs-CM-T pre-neutralized with either CCL17 or CCL22 antibody (lines 3 and 4, Fig. 3J). Strikingly, pre-incubation with both antibodies entirely blocked the GRP78 upregulation (line 5, Fig. 3J).

As CCR4 is the shared receptor for both CCL17 and CCL22 and is reported to be overexpressed in tumor cells [28, 29], we next examined its expression on the membrane of CRC cells and reproducibly detected strong signals in the isolated membrane fractions (Fig. S4C). Next, to investigate whether the CCL17/CCL22-CCR4 interaction is mainly responsible for GRP78 induction in CRC cells, we pre-neutralized the CRC cancer cells with either CCR4 antibody or its antagonist, and found that TAMs-CM-T failed to induce GRP78 upregulation under both conditions (lines 6 and 8, Fig. 3J). Moreover, such CCL17/CCL22-CCR4 pair interaction dependency was also recapitulated in DLD1 cells treated with 20% TAMs-CM-H (Fig. 3K). Taken together. these data indicated that the intercellular ligand-receptor interaction between CCL17/CCL22 (secretome of TAMs) and CCR4 (CRC tumors) mainly mediated the expression of GRP78 in tumor cells.

### ER-associated ATF6 Activation and calcium spherical aggregation are required for TAMs-induced GRP78 upregulation

**Fig. 4:**
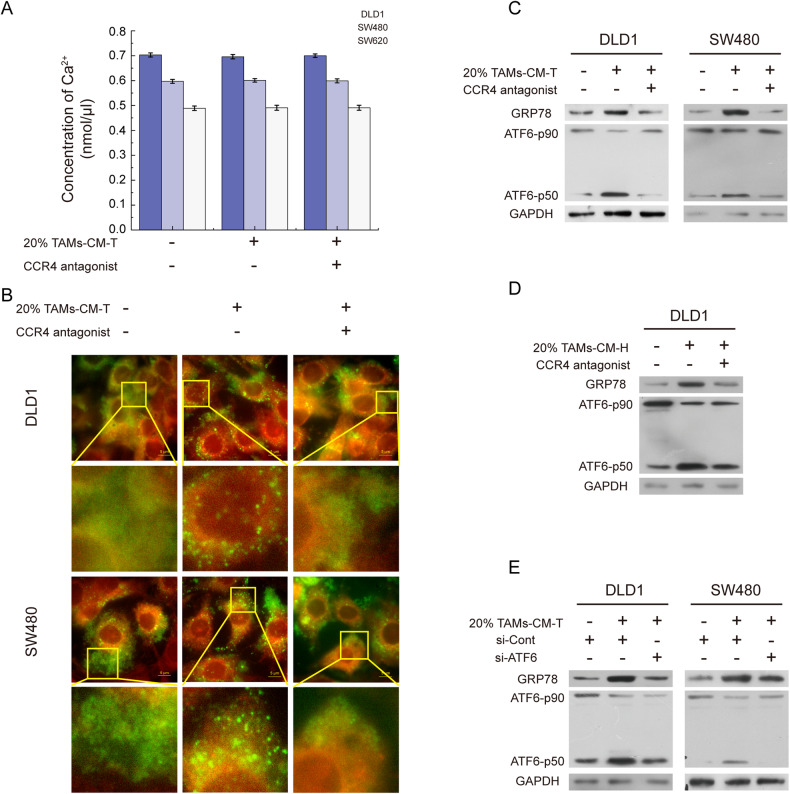
The CCL17/CCL22-CCR4 pathway leads to the activation of ATF6 by inducing calcium spherical aggregation in ER. **A** The bar graph represented the intracellular total calcium concentration in DLD1, SW480, and SW620 cells incubated with 20% M2-CM-T or/and CCR4 antagonist. **B** A fluorescence stain was applied to detect the distribution of calcium in ER, Fluo-3 AM marked calcium (green), and ER-tracker marked ER (red). **C** The level of GRP78 and ATF6-p50 were detected in DLD1 and SW480 cells treated with 20% TAMs-CM-T or/and CCR4 antagonist. **D** The level of GRP78 and ATF6-p50 were detected in DLD1 cells treated with 20% TAMs-CM-H or/and CCR4 antagonist. **E** After knocking down ATF6, the expression of GRP78 and the activation of ATF6 were detected in DLD1 and SW480 with 20% TAMs-CM-T treatment.

GRP78 is the major sensor of ER calcium homeostasis and ER stress, and the abnormal expression of GRP78 is closely associated with the disturbance of ER calcium homeostasis [28]. To dissect whether calcium homeostasis is involved in TAMs-CM-induced GRP78 expression, we first examined the intracellular levels of total calcium and found no obvious difference between the control group and the TAMs-CM-T-treated group regardless of CCR4 blockage (Fig. 4A). Interestingly, further imaging analysis identified TAMs-CM-T treatment led to the aggregation of calcium in ER, which was impaired by the pretreatment of tumor cells with CCR4 antagonist (Fig. 4B). Previous studies showed that the disturbance of ER calcium homeostasis induces ATF6 cleavage and facilitates the binding of ATF6-p50 (the active form of ATF6) to the promoter region of GRP78 [29, 30]. Western blotting results revealed that TAMs-CM treatment led to the co-occurrence of increased GRP78 and ATF6-p50, and further CCR4 antagonist treatment reversed such effect (Fig. 4C, D), which resembled the pattern of calcium aggregation (Fig. 4B). Furthermore, blocking the CCL17/CCL22-CCR4 signal axis or knockdown of *ATF6* led to the decrease of ATF6-p50 and subsequent the inhibition of GRP78 expression (Fig. 4E). These results suggested that CCL17/CCL22-CCR4 axis was required for TAMs-CM to communicate with CRC cells by activating aggregation of ER calcium, and the ER calcium aggregation and ATF6 activation were the key upstream events of GRP78 upregulation that induced by TAMs-CM treatment.

### Inactivation of IP3R initiates abnormal calcium distribution in ER

**Fig. 5:**
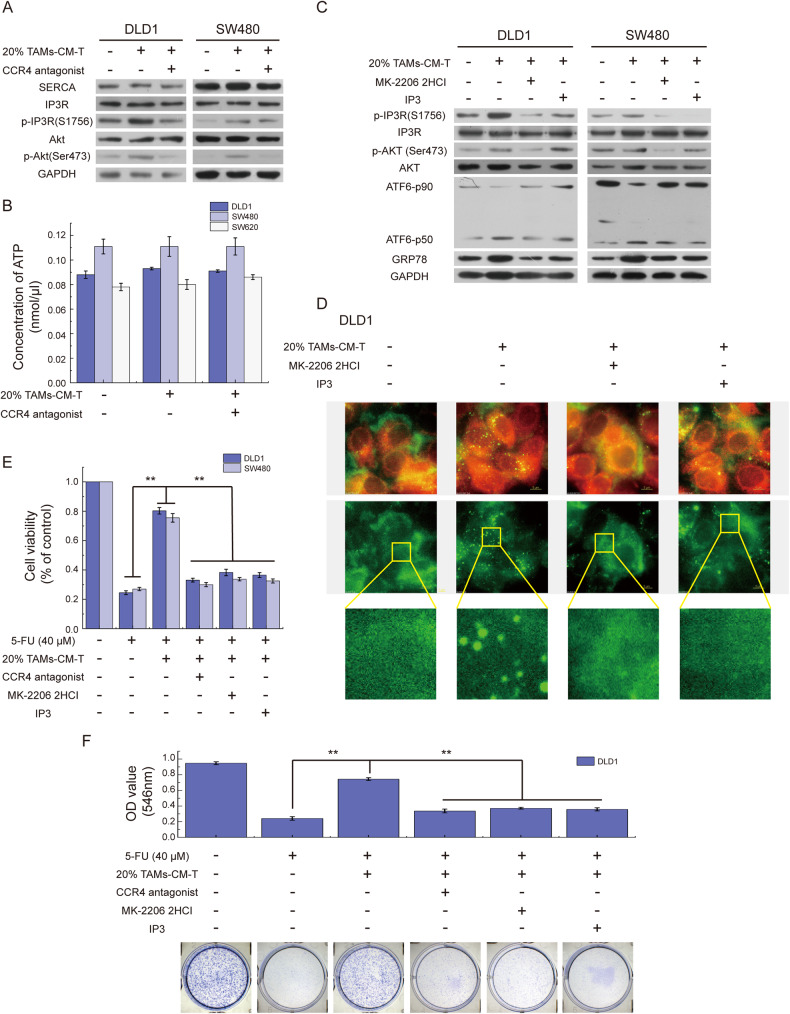
IP3R inactivation induces the abnormal calcium distribution. **A** SERCA, IP3R, p-IP3R, Akt, and p-Akt were detected by western blot in DLD1 and SW480 cells treated with 20% TAMs-CM-T or/and CCR4 antagonist. **B** The bar graph represented the intracellular total ATP concentration of DLD1, SW480, and SW620 cells treated with 20% TAMs-CM-T or/and CCR4 antagonist. **C** After treatment with MK-2206 2HCI, IP3, and 20% TAMs-CM-T, the expression of IP3R, p-IP3R, Akt, p-Akt, ATF6-p50, and GRP78 were detected by western blot in DLD1 and SW480. **D** After treatment with 20% TAMs-CM-T, MK-2206 2HCI, and IP3, DLD1 cells were fluorescence stained with Fluo-3 AM (green) and ER-tracker (red). **E** After blocking the CCL17/CCL22-CCR4 pathway with a CCR4 antagonist or activating IP3R with MK-2206 2HCI and IP3, the effect of TAMs-CM-T on the decreases of DLD1, SW480, and SW620 cell viability induced by 5-FU was detected by MTT, **p < 0.01. **F** After blocking the CCL17/CCL22-CCR4 pathway with a CCR4 antagonist or activating IP3R with MK-2206 2HCI and IP3, the effect of 20% TAMs-CM-T on the diminution of DLD1 cell colony formation capacity induced by 5-FU was detected by Colony-Formation Assay, **p < 0.01.

ER calcium flux is regulated by sarco/endoplasmic reticulum Ca^2+^-ATPase (SERCA, transports calcium from the cytosol into ER) and inositol 1,4,5-trisphosphate receptor (IP3R, releases calcium from the ER store) [31, 32]. The activity of SERCA is mainly influenced by intracellular ATP concentration [31, 33], whereas the activity of IP3R is up-regulated by its ligand inositol 1,4,5-trisphosphate (IP3) and down-regulated through phosphorylation by phosphorylated-AKT [34]. To explore whether TAMs-CM treatment affects the calcium transport through SERCA and IP3R, we determined protein levels of SERCA and IP3R and phosphorylated IP3R in TAMs-CM treated DLD1 and SW480 cells. Despite no change in SERCA and IP3R expression, phosphorylation of IP3R and AKT were markedly increased upon TAMs-CM-T treatment, which was impaired via further inhibition of the CCL17/CCL22-CCR4 signaling axis (Fig. 5A). Nevertheless, the intracellular ATP detection revealed that the SERCA activity was not been impacted by TAMs-CM (Fig. 5B).

Next, we employed AKT inhibitor (MK-2206 2HCI) and IP3 (a ligand of IP3R) to modulate IP3R activation status, and examined the impact on corresponding GRP78 expression and calcium distribution. As expected, IP3 treatment inhibited IP3R phosphorylation and activated IP3R without influencing the phosphorylation of AKT (line 4, Fig. 5C), whereas MK-2206 2HCI treatment simultaneously suppressed the phosphorylation of AKT and IP3R (line 3, Fig. 5C). Nevertheless, both MK-2206 2HCI and IP3 efficiently abolished TAMs-CM-induced ATF6 cleavage and GRP78 upregulation (Fig. 5C), as well as the ER calcium aggregation (Fig. 5D). More importantly, consistent with the requirement of GRP78 in assisting CRC 5-FU resistant, disruption of CCL17/CCL22-CCR4 signal axis by the antagonist or treatment of MK-2206 2HCI and IP3 all efficiently rescued 5-FU resistance of tumors cells as measured by both cell viability assay (Fig. 5E) and colony-formation assay (Fig. 5F).

Taken together, these results indicated that chemokines CCL17/CCL22 within TAMs-CM interact with CCR4 of CRC cells, which facilitates the inactivation of IP3R, and results in the calcium aggregation in ER and GRP78 upregulation.

### Excessive GRP78 facilitates the membrane translocation of MRP1

**Fig. 6:**
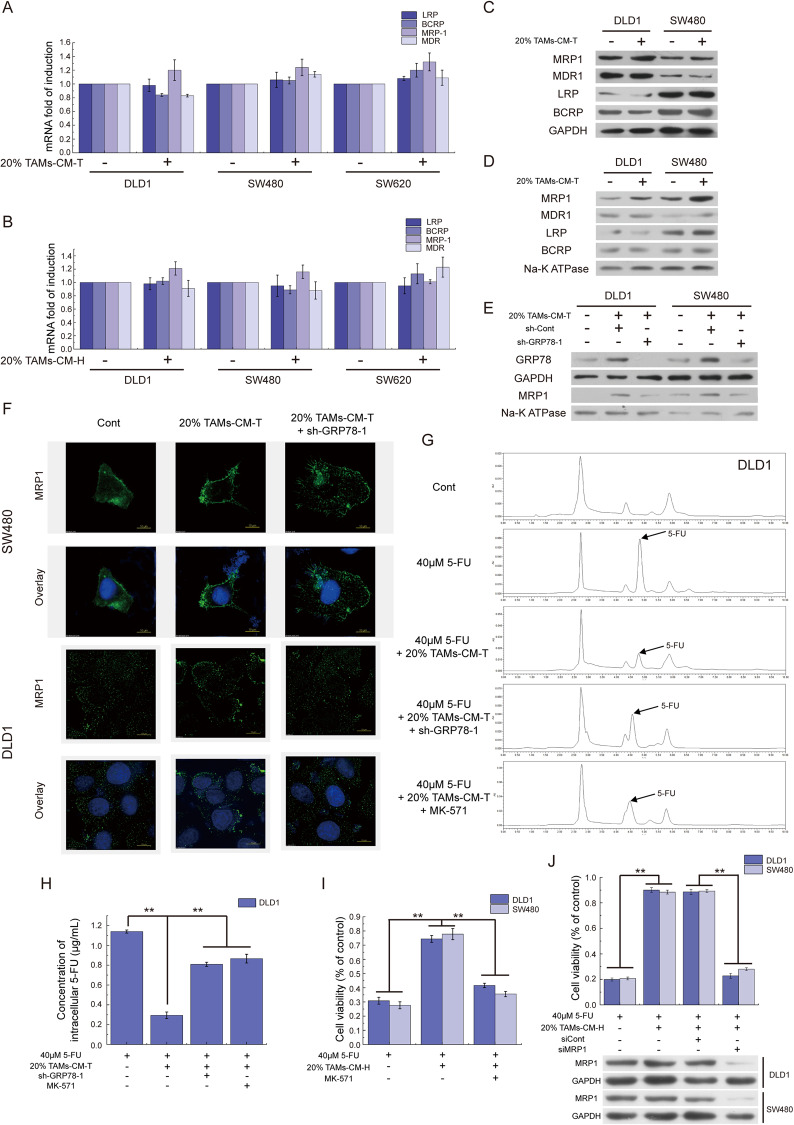
Excessive GRP78 facilitates the membrane translocation of MRP1. **A** QRT-PCR was applied to detect the expression of transports (LRP, BCRP, MRP-1, and MDR) in DLD1, SW480, and SW620 cells which were treated with 20% TAMs-CM-T. **B** QRT-PCR was applied to detect the expression of transports (LRP, BCRP, MRP-1, and MDR) in DLD1, SW480, and SW620 cells which were treated with 20% TAMs-CM-H. **C** Western blot was applied to detect the expression of transports in DLD1 and SW480 cells treated with 20% TAMs-CM-T. **D** After isolating the cytomembrane, the western blot was applied to detect the membrane translocation of the transports in DLD1 and SW480 cells treated with 20% TAMs-CM-T. **E** Knocking down GRP78, the membrane translocation of MRP1 was detected by western blot in DLD1 and SW480 cells which were treated with 20% TAMs-CM-T. **F** Knocking down GRP78, the membrane translocation of MRP1 was detected by immunofluorescence in DLD1 and SW480 cells which were treated with 20% TAMs-CM-T. **G** After knocking down GRP78 by shRNA, or inhibiting MRP1 by MK-571, 20% TAMs-CM-T were applied to evaluate its effects on the 5-FU efflux of DLD1 by HPLC. **H** The bar graph represented the concentration of 5-FU in DLD1 cells that were shown in G, **p < 0.01. **I** After inhibiting MRP1 by MK-571, the viability of cells was detected in DLD1 and SW480 cells which were treated with 20% TAMs-CM-H, **p < 0.01. **J** After knockdown MRP1 by siRNA, the viability of cells was detected in DLD1 and SW480 cells which were treated with 20% TAMs-CM-H, **p < 0.01.

After characterization of the upstream regulatory network for CCL17/CCL22-induced GRP78 upregulation, we next moved on to dissect how GRP78 dysregulation endows 5-FU resistant in CRC. Due to overexpression and translocation of the drug effluxes transporters such as LRP, BCRP, MRP1, and MDR are the primary causes of chemotherapy resistance of tumors [35], we first examined their expression and found no obvious changes in both the mRNA (Fig. 6A, B) and protein levels expression (Fig. 6C) upon TAMs-CM treatment. Because these transporters always exert their functions within the cytomembrane, we further isolated cell membrane proteins and compared the membrane-associated protein level changes. Unexpectedly, this analysis showed that 20% TAMs-CM-T stimulation specifically facilitated MRP1, but not LRP, BCRP, and MDR, translocation to cytomembrane (Fig. 6D). Moreover, *GRP78* knockdown impaired the TAMs-CM-T-induced MRP1 translocation, suggesting that elevated GRP78 acts as the driver of MRP1 translocation (Fig. 6E). Indirect immunofluorescence also revealed that 20% TAMs-CM-T promoted aggregation of MRP1 on the cell membrane, while *GRP78* knockdown reversed this effect in both SW480 and DLD1 cells (Fig. 6F).

To further evaluate the role of TAMs secretome, GRP78 upregulation, and MRP1 translocation in modulating 5-FU drug resistance in CRC cells, we next applied HPLC to detect the intracellular 5-FU level after 8 h of treatment with 5-FU. The results showed that 20% TAMs-CM-T significantly reduced the 5-FU concentration in DLD1 cells compared with the control group (Fig. 6G). However, either antagonizing GRP78 by shRNA, or inhibiting MRP1 by chemical molecule inhibitor (MK-571) or siRNA, led to the increased intracellular concentration of 5-FU (Figs. 6G, H and S5A, B). MTT assay further revealed that inhibition of MRP1 by MK-571 or siRNA reversed the promoting effect of 20% TAMs-CM on 5-FU resistance of tumor cells (Figs. 6I, J and S5C, D). These data indicated that the membrane translocation of MRP1 was responsible for TAM secretome-induced 5-FU resistance of CRC cells, and GRP78 plays a critical role in the translocation of MRP1.

### GRP78 interacts with MRP1 and co-translocates to cytomembrane

**Fig. 7:**
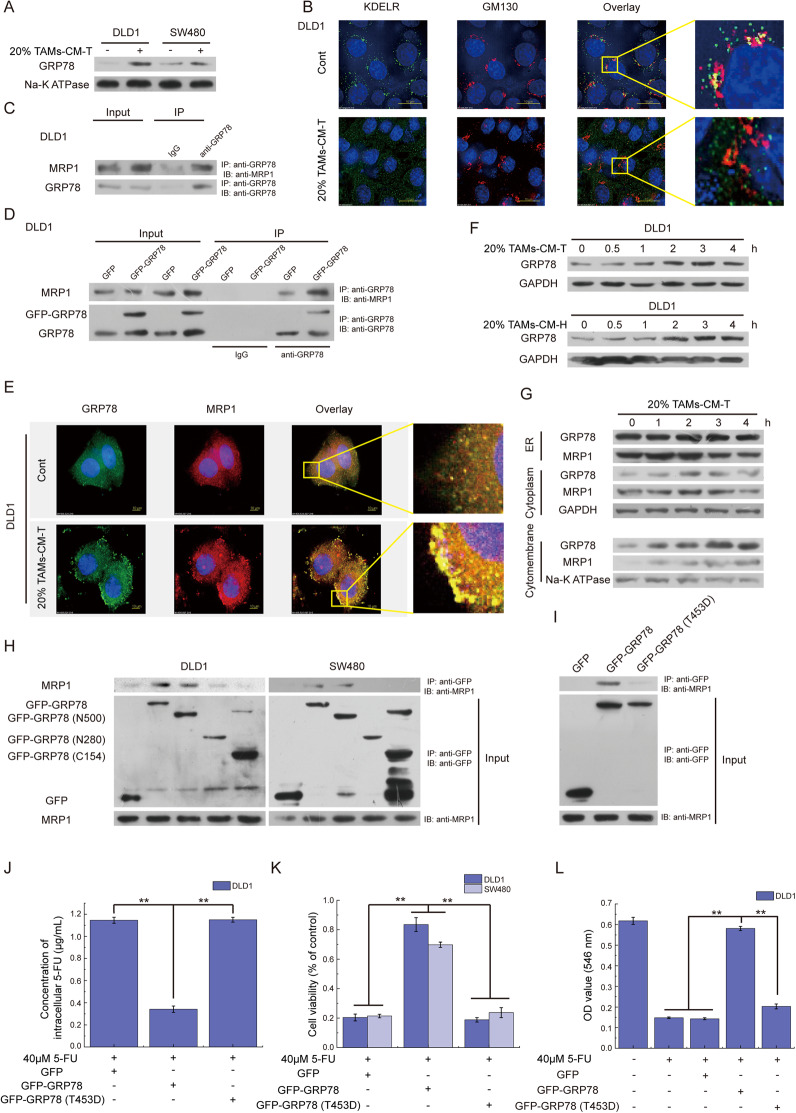
GRP78 interacts and co-translocates with MRP1 to cytomembrane. **A** After isolating the cytomembrane, the membrane translocation of GRP78 was detected by western blot in DLD1 and SW480 cells treated with 20% TAMs-CM-T. **B** The detachment of the KDEL receptor (KDELR) from Golgi was detected by immunofluorescence stain, KDELR (green), GM130 marked Golgi (red). **C** The interaction of GRP78 and MRP1 was detected by Co-IP in DLD1 cells. **D** The interaction of GRP78 and MRP1 was detected by Co-IP in GRP78-overexpressed DLD1 cells. **E** With 20% TAMs-CM-T treatment, the colocation of GRP78 and MRP1 was detected by immunofluorescence stain, GRP78 (green), MRP1 (red). **F** With 20% TAMs-CM-T and 20% TAMs-CM-H treatment, the expression of GRP78 was detected by western blot according to the gradient of time. **G** With 20% TAMs-CM-T treatment, the expression of GRP78 and MRP1 in ER, cytoplasm, and cytomembrane were detected according to the gradient of time. **H** GRP78 (T500), GRP78 (T280), and GRP78 (C154) were overexpressed in DLD1 cells, and the Co-IP was applied to detect the interaction of MRP and deletion mutants of GRP78. **I** GRP78 (WT) and GRP78 (T453D) were overexpressed in DLD1, and the Co-IP was applied to detect the interaction of MRP and GRP78 (T453D). **J** After overexpressing GRP78 (WT) and GRP78 (T453D), the 5-FU in DLD1 was detected by HPLC, **p < 0.01. **K** After overexpressing GRP78 (WT) and GRP78 (T453D), the viability of the cell was detected in DLD1 and SW480, which were treated with 40 μM 5-FU, **p < 0.01. **L** After overexpressing GRP78 (WT) and GRP78 (T453D), the formation of the cell colony was detected in DLD1 cells, which were treated with 40 μM 5-FU, **p < 0.01.

After shown that excessive GRP78 promoted the membrane translocation of MRP1, we continued to explore the mechanism underlined to drive this process. It is reported that the microenvironment stress in the tumor resulted in the inadvertent transport of excessive GRP78 to the cell surface along with its client proteins [36]. Therefore, we hypothesized that the MRP1 acts as one of the GRP78 clients, and excessive GRP78 promoted membrane translation of MRP1. Indeed, more GRP78 was detected in the cell membrane after 20% TAMs-CM-T treatment (Fig. 7A). Due to the detachment of the KDEL receptor (KDELR) from the Golgi is required for GRP78 translocation [37], the location of KDELR was detected with TAMs-CM-T treatment. In the control group, KDELR was co-located with GM130-marked Golgi, in contrast, KDELR was not co-located with Golgi and dispersed in the cytoplasm after 20% TAMs-CM-T treatment (Fig. 7B). These results indicated that 20% TAMs-CM-T facilitated the escape of GRP78 from the Golgi-to-ER recycle system. We next detected the interaction of GRP78 and MRP1. Co-IP revealed that GRP78 was able to interact with MRP1 (Fig. 7C), and the interaction became more obvious when 20% TAMs-CM-T treatment (Fig. S6A) or GRP78 overexpression (Fig. 7D). Immunofluorescence also revealed the membrane translocation of excessive GRP78 (simulated by overexpressing GFP-GRP78, Fig. S6B), and the co-location of MRP1 and GRP78 on the cytomembrane after 20% TAMs-CM-T treatment (Fig. 7E). To further verify our speculation, with 20% TAMs-CM-T treatment, the dynamic location of GRP78 and MRP1 were detected. Western blotting revealed that protein levels of total GRP78 and cytomembrane GRP78 were gradually increased with time, however, GRP78 of cytoplasm peaked after 2 h of 20% TAMs-CM-T treatment, and then gradually decrease, while no significant change in GRP78 was observed in ER (Fig. 7F, G). Translocation of cytoplasm MRP1 was similar to that of GRP78, and the level of MRP1 in ER decreased gradually with time (Fig. 7G). These results indicated that excessive GRP78 bound MRP1 and co-translocated to the cytomembrane.

To further identify the interaction sites of GRP78 with MRP1, the three deletion mutants of GRP78 were constructed as Fig. S6C. The results of Co-IP showed that MRP1 was detected in the complex of GRP78-T500 besides full-length GRP78, which indicated the peptide-binding domain of GRP78 is required for the interaction of GRP78 and MRP1 (Fig. 7H). Threonine at position 453 is crucial for the activity of the GRP78 peptide-binding domain, and the T453D mutation prevents GRP78 from binding to protein substrates [38]. As expected, T453D mutation impaired the interaction between GRP78 and MRP1 (Fig. 7I), and GRP78 (T453D) failed to promote the efflux of 5-FU and restore cell viability (Figs. 7J, K and S6D). Moreover, the mutation of T453D made GRP78 lose its ability to promote tumor colony formation (Figs. 7L and S6E). All these results suggested that excessive GRP78 interacted and co-translocated with MRP1 to cytomembrane, and the residue threonine 453 was required for GRP78 to interact with MRP1.

### GRP78 is essential for TAM-associated 5-FU resistance of CRC tumor in vivo

**Fig. 8:**
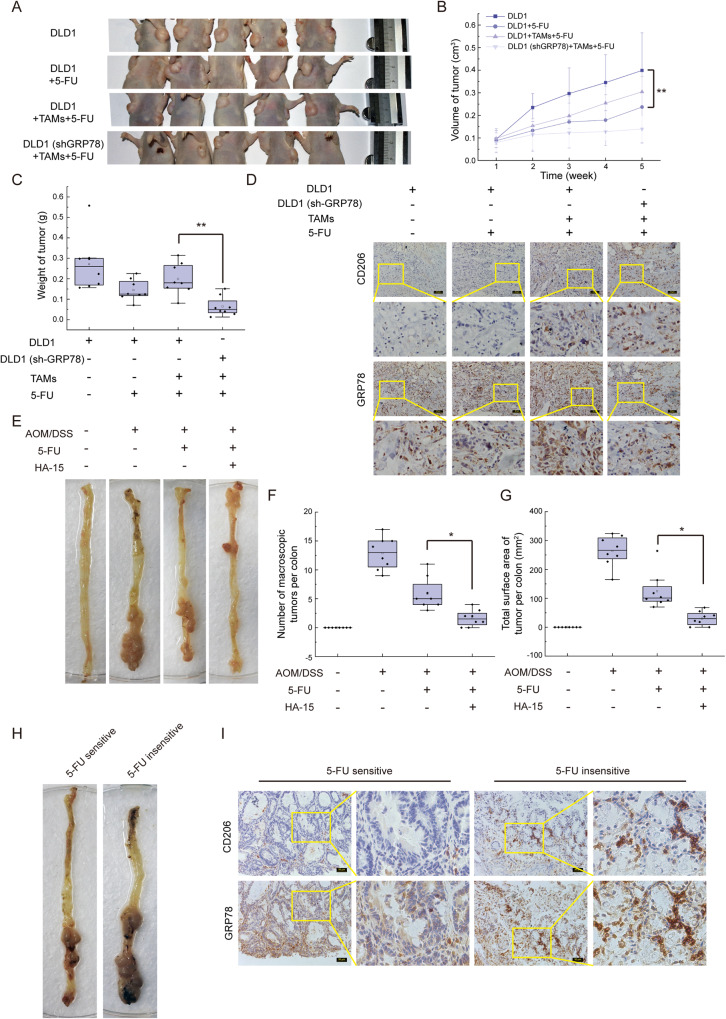
GRP78 is required for M2 macrophages to promote 5-FU resistance of tumor cells in vivo. **A** Photographs of tumors in mice after being administered with 5-FU. The tumor was formatted with DLD1 cells, DLD1 cells + TAMs, and DLD1 (shGRP78) cells + TAMs, respectively. **B** The volume of tumors was measured once a week during the experiment, **p < 0.01. **C** Mass of dissected tumors, **p < 0.01. **D** Immunohistochemical staining with antibodies against GRP78 and CD206 was performed on the dissected tumors. **E** The photo of macroscopic colon tumors induced by AOM/DSS. **F** The number of macroscopic tumors (tumors larger than 1 mm in diameter), *p < 0.05. **G** The tumor burden of AOM/DSS induced mice, *p < 0.05. **H** The macroscopic findings of 5-FU sensitive and insensitive tumors. **I** The expression of GRP78 and the infiltration of TAMs were detected by immunohistochemical in 5-FU sensitive and insensitive tumors that showed in **H**.

To further determine the role of GRP78 in TAMs-mediated 5-FU resistance, both DLD1 cells and TAMs were injected into the subcutis of immunocompromised (nu/nu) mice as illustrated in Fig. S7A. Intraperitoneal injection of 5-FU inhibited the growth of xenograft tumors, and the inhibition of 5-FU on tumor growth was impaired when TAMs inoculated with DLD1, however, knocking down *GRP78* increased the sensitivity of the tumor to 5-FU even in the presence of TAMs (Fig. 8A–C). The results of immunohistochemistry displayed that the presence of TAMs (marked with CD206) indeed led to high expression of GRP78 (Fig. 8D). These findings indicated that TAMs take an active role in the 5-FU resistance of tumors, in which GRP78 was required.

HA15 is a molecule that targets GRP78 specifically, which further leads to the dissociation of GRP78 from its partners [39]. Indeed, in the presence of HA15, the interaction of GRP78 and MRP1 decreased (Fig. S7B). Then HA15 was used with 5-FU on AOM/DSS-induced colon tumors. Although 5-FU suppressed the colonic tumorigenesis, the combination of 5-FU and HA15 further reduced the multiplicity of colonic tumors (Fig. 8E, F) and tumor burden (Fig. 8G) significantly. Remarkably, the tumor of one mouse was significantly larger than that of other mice in the 5-FU single treatment group, which indicated the mouse was insensitive to 5-FU (Fig. 8H). Immunohistochemical results showed that more infiltration of M2 macrophages was detected in the 5-FU-insensitive tumor, and expression of GRP78 was upregulated in the tumor cells adjacent to TAMs (Fig. 8I). All these findings indicated that TAMs and GRP78 were required for 5-FU resistance of tumor cells, and HA15 plus 5-FU was expected to be a new anti-tumor strategy.

## Discussion

GRP78 is an important ER stress signaling sensor and regulator, its overexpression is always attributed to microenvironmental stress [40], whether any other factors activate the expression of GRP78 in tumor cells. In this study, we identified two chemokines (CCL17 and CCL22) that played an important role in activating the expression of *GRP78*. CCL17 and CCL22 as two of the major products of TAMs share a common receptor CCR4 that typically exists on the tumor cell surface [41]. Activated CCR4 can activate PI3K/AKT axis [42], and phosphorylated AKT functions as a negative regulator of IP3R activity [34]. It is known that IP3R mediates calcium efflux from ER [32], the inactivation of IP3R leads to the imbalance of calcium and high expression of GRP78. Combining our results and these established findings indicated a new axis of CCL17/22-CCR4-PI3K/AKT-IP3R rather than microenvironment stress led to the high level of GRP78 in tumor cells.

PI3K/AKT axis possesses numerous downstream targets. One of these targets is the IP3R, which plays an important role in releasing calcium from the ER store and maintaining ER calcium homeostasis [43]. Phosphorylation of IP3R by kinase PKB/AKT inhibits its activity. However, IP3 (the ligand of IP3R) activates IP3R, which promotes the release of calcium from ER and relieves ER stress. More importantly, there is a key factor, phosphatidylinositol [4, 5]-bisphosphate (PIP2), which can be transformed into phosphatidylinositol [3, 4, 5]-trisphosphate (PIP3, the activator of AKT) by PI3K, and it can also be hydrolyzed into IP3 by hormone-sensitive phospholipase C (PLC) enzymes [44]. Therefore, we speculated that the TAMs-induced CCL17/CCL22-CCR4 pathway enhanced the activation of PI3K. Activated-PI3K possibly competed for substrate (PIP2) with PLC and resulted in high PIP3 and low IP3, which eventually reduced the activity of IP3R via two paths.

Calcium is an important second messenger and is required for many pathways in cells [45]. ER is the main intracellular calcium store, and the total calcium in the ER is much higher than in cytosol [45]. In our study, TAMs-CM treatment induced the inactivation of IP3R, which was bound to suppress the release of calcium from the ER to the cytosol. Although the decrease in cytoplasmic calcium was not observed, the calcium aggregation could be the result of calcium accumulation in the ER. It has been reported that expression of GRP78 is markedly increased in response to depletion of the ER calcium pool, but the calcium released via the IP3R can transfer into mitochondria, leading to mitochondrial-related autophagy and apoptosis [46], which conflicts with the cellular protection role of GRP78. Our study suggested that TAMs-CM treatment inactivated IP3R, which not only increased expression of *GRP78* but also kept low calcium levels in mitochondrial and maintained mitochondrial bioenergetics, thereby facilitating tumor progress.

**Fig. 9:**
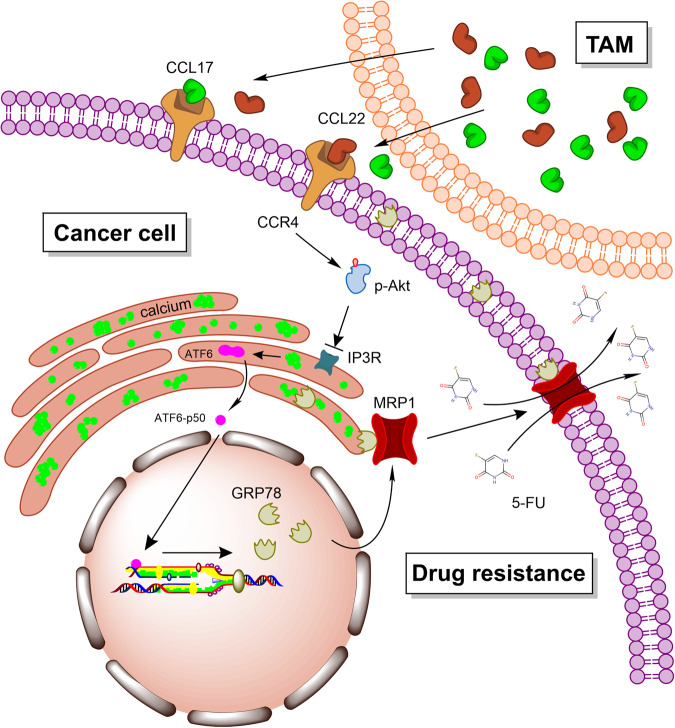
Working model. CCL17 and CCL22 which were secreted by TAMs activated the PI3K/AKT pathway in tumor cells via CCR4. Specifically, AKT phosphorylation inactivated IP3R, and induced calcium aggregation in the ER, which further activated ATF6 and increased GRP78 expression. Excessive GRP78 bond with MRP1 and pulled it on cytomembrane, therefore, the membrane translocation of MRP1 was the direct cause of TAMs-CM-induced 5-FU efflux and resistance.

To date, one major reason for the failure of cancer therapy is drug resistance. Drug resistance is involved in a variety of factors, and the most common reason for the acquisition of resistance to anticancer drugs is dysregulated expression and membrane translocation of the energy-dependent transporters. Both TAMs and GRP78 had been reported to play important roles in tumor drug resistance. TAMs are involved in tumor-promoting inflammation, avoiding immune destruction, and inducing angiogenesis to promote tumor drug resistance [17]. GRP78 was reported to reduce chemotherapy sensitivity by assisting tumor cells to resist apoptosis [47, 48]. However, few studies provide immediate cues about TAMs and GRP78 in regulating drug transporters that mediate drug resistance. In this study, we found that TAMs promoted the expression of *GRP78*, and excessive GRP78 interacted with MRP1 and further moved MRP1 to the tumor cell membrane, which increased the efficiency of drug efflux. In summary, our findings showed the correlation among TAMs, GRP78, and MRP1, providing direct evidence that TAMs and GRP78 are important regulators of tumor drug resistance (Fig. 9).

## Supplementary information

Original data

Supplementary Figures and legends
