# Supplementary material for: Tumor-associated macrophages confer colorectal cancer 5-fluorouracil resistance by promoting MRP1 membrane translocation via an intercellular CXCL17/CXCL22–CCR4–ATF6–GRP78 axis
Source: Cell Death Dis. 2023 Sep 1;14(9):582. doi: 10.1038/s41419-023-06108-0 (PMC10474093; doi:10.1038/s41419-023-06108-0)
Supplement: Supplementary file 1 — Original data [file 41419_2023_6108_MOESM1_ESM.pdf]

Fig 2E

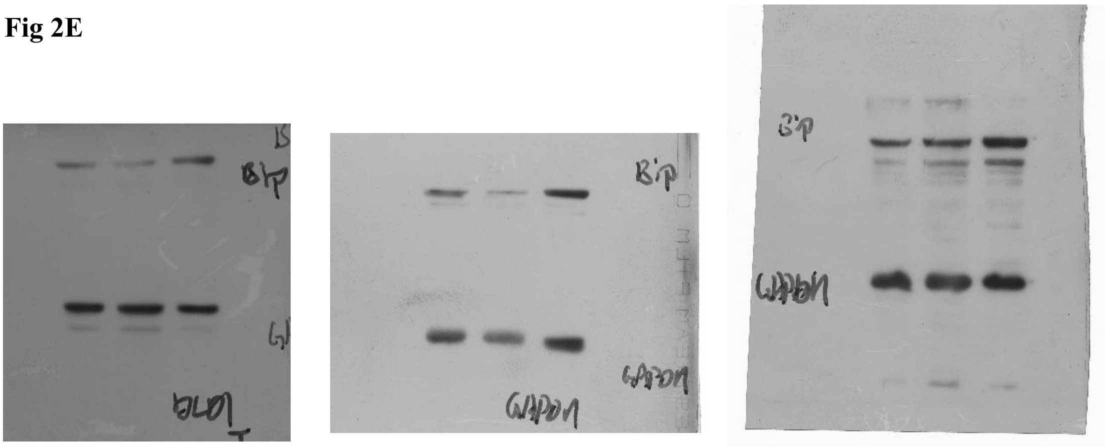

DLD1 SW480 SW620

Fig 2F

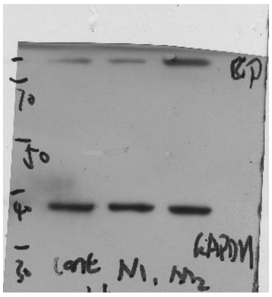

Fig 2G

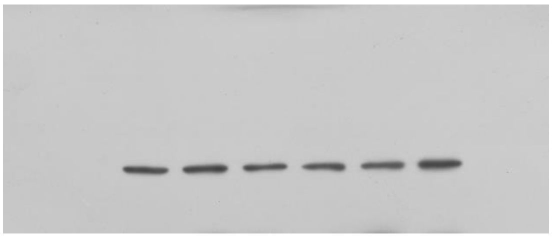

GAPDH

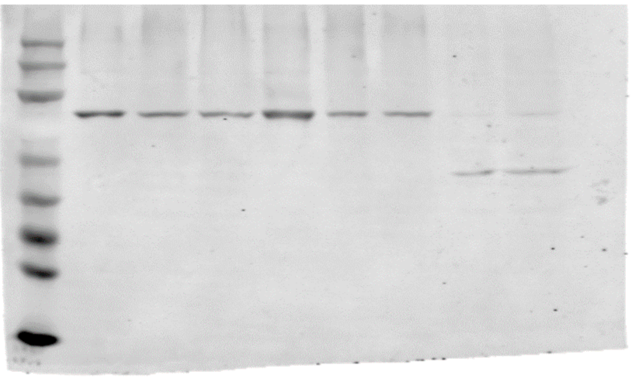

Fig 3E

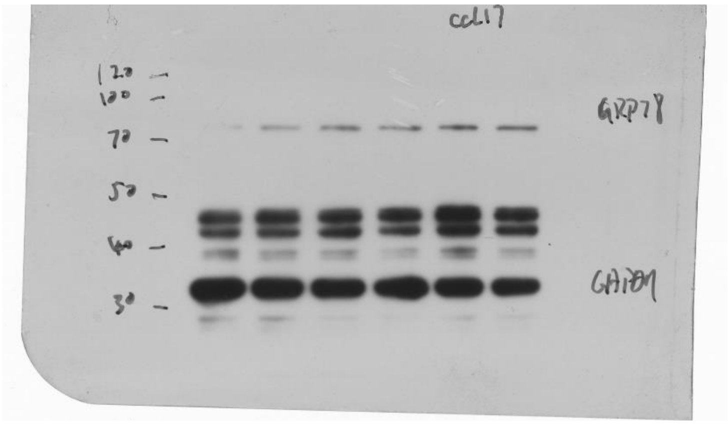

CCL17

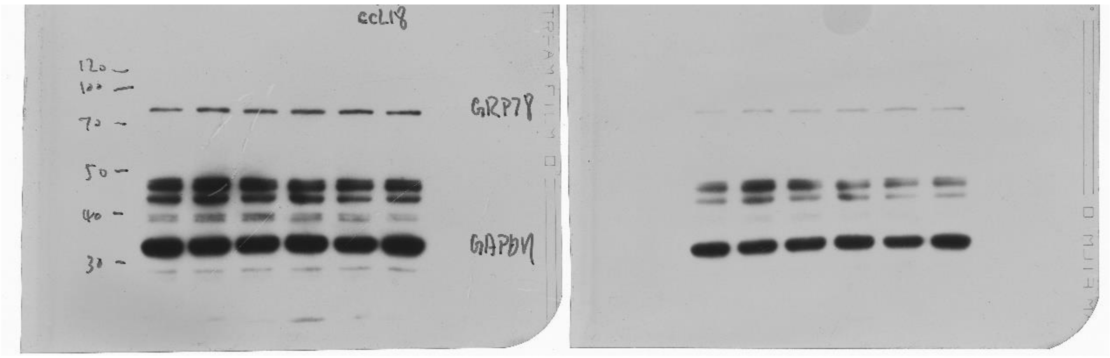

CCL18

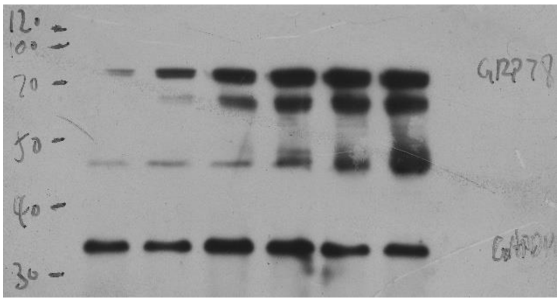

CCL22

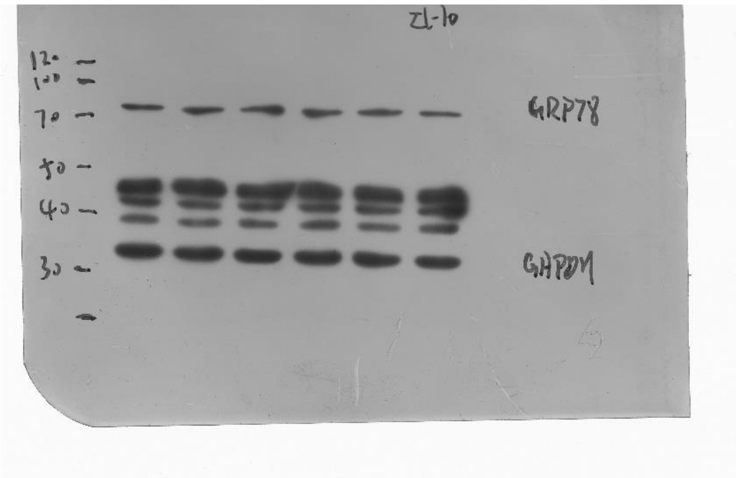

IL-10

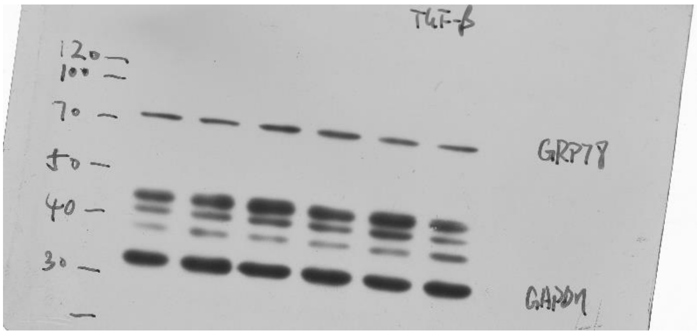

TGF-β

Fig 3F

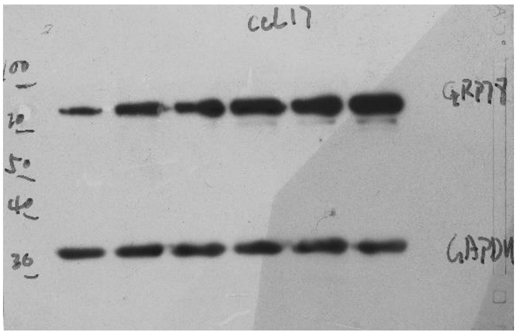

CCL17

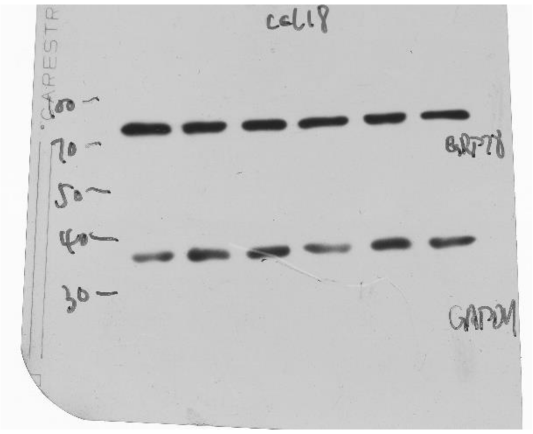

CCL18

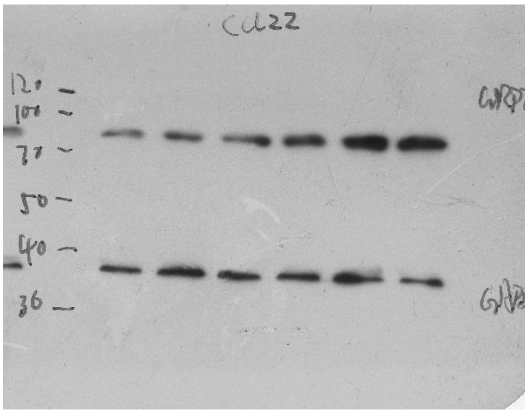

CCL22

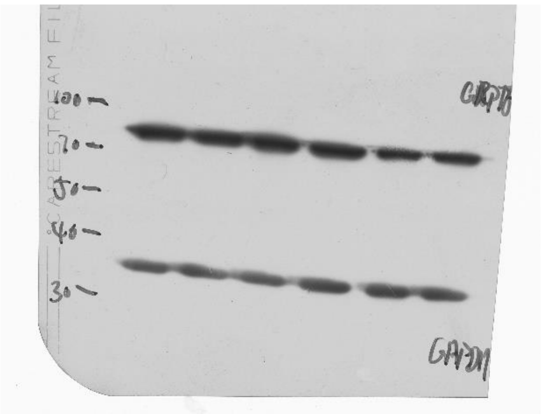

IL-10

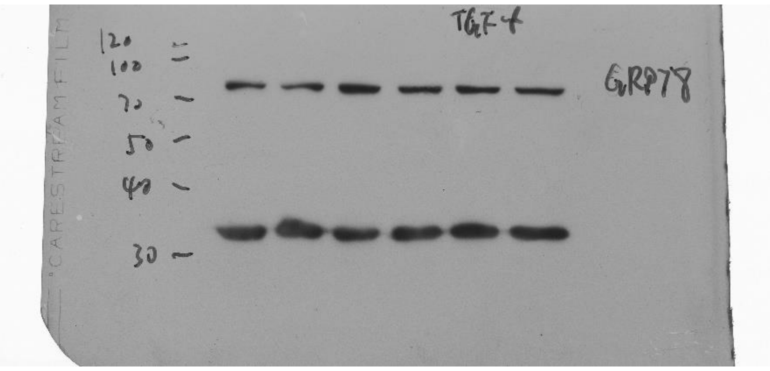

TGF-β

Fig 3G

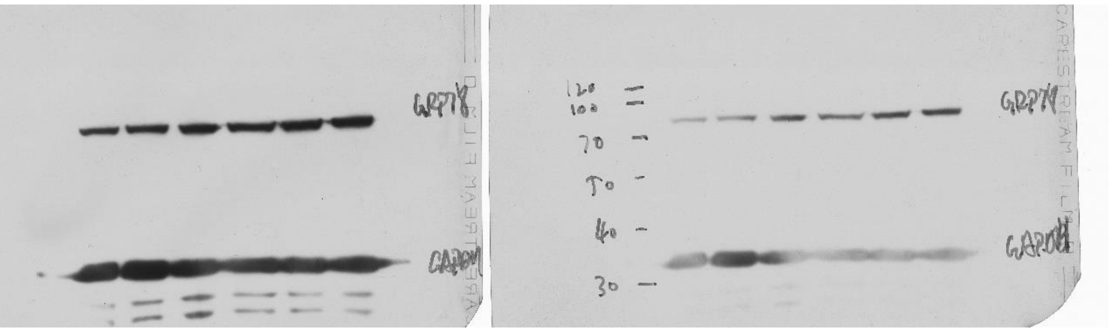

CCL17

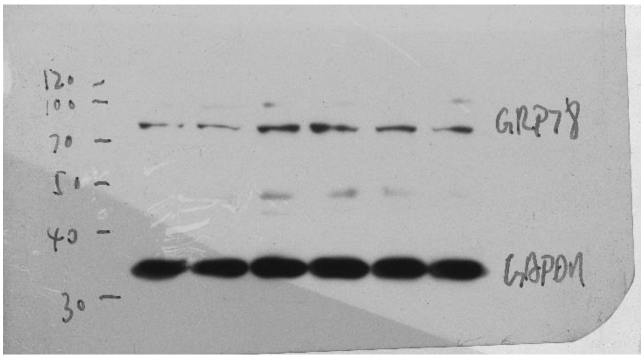

CCL22

Fig 3J

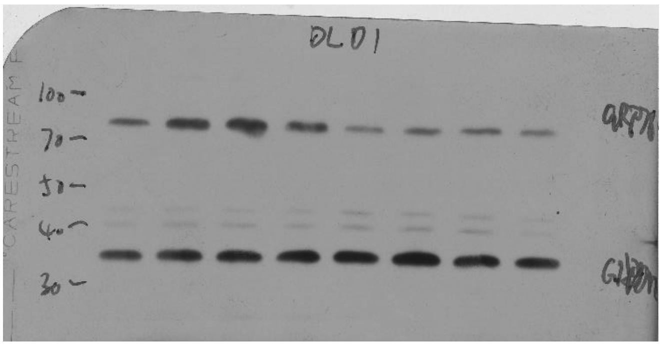

DLD1

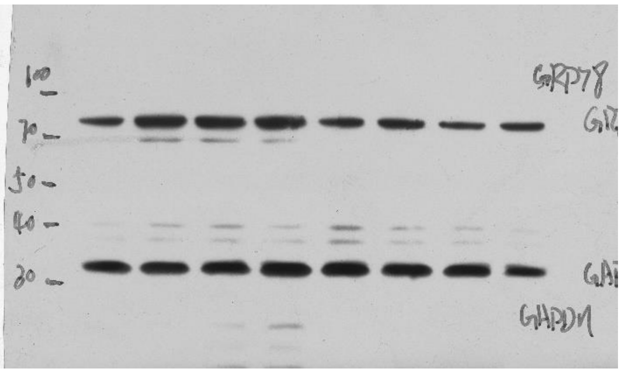

SW480

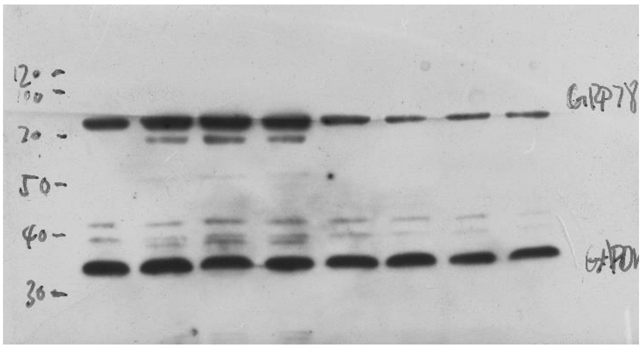

SW620

Fig 3K

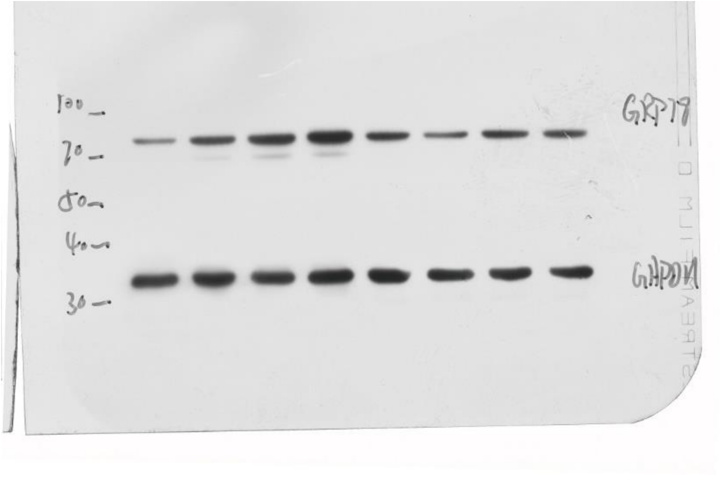

Fig 4C

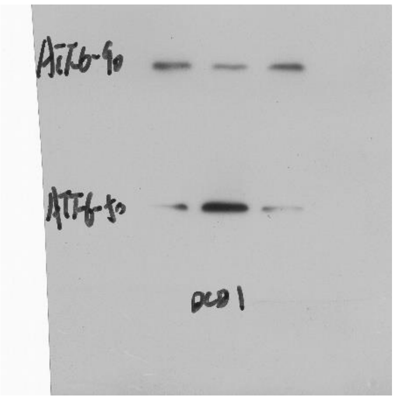

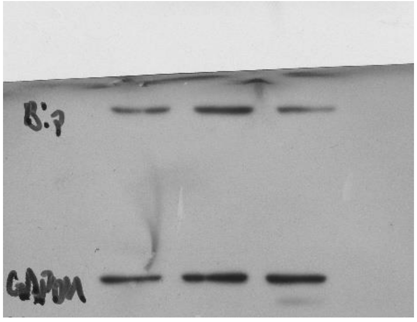

DLD1-ATF6

DLD1-GRP78-GAPDH

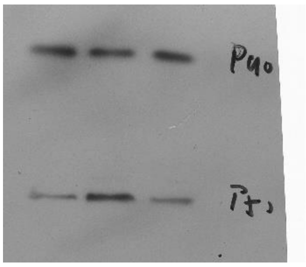

SW480-ATF6

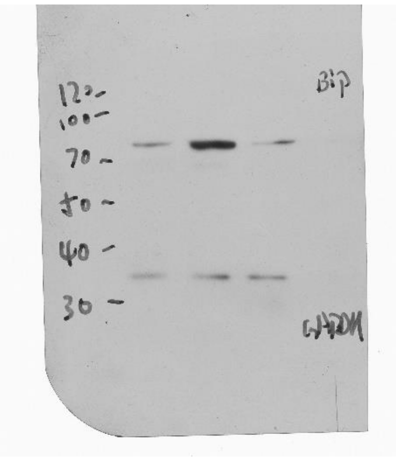

SW480-GRP78-GAPDH

Fig 4D

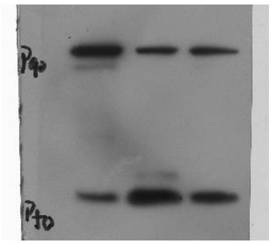

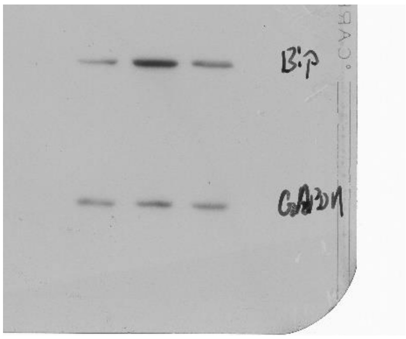

ATF6

GRP78-GAPDH

Fig 4E

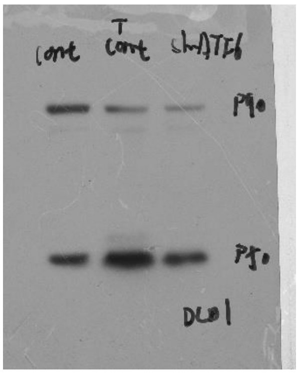

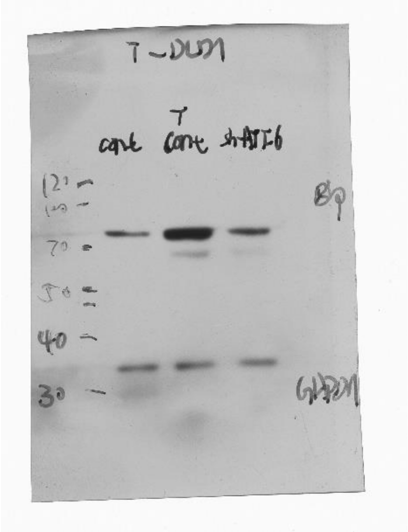

DLD1-ATF6

DLD1-GRP78-GAPDH

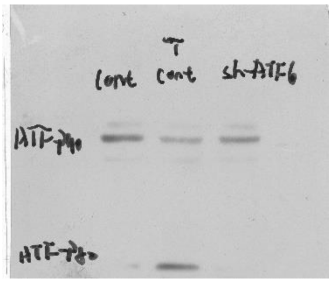

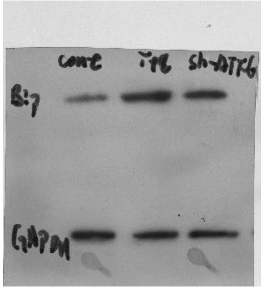

SW480-ATF6

SW480-GRP78-GAPDH

Fig 5A

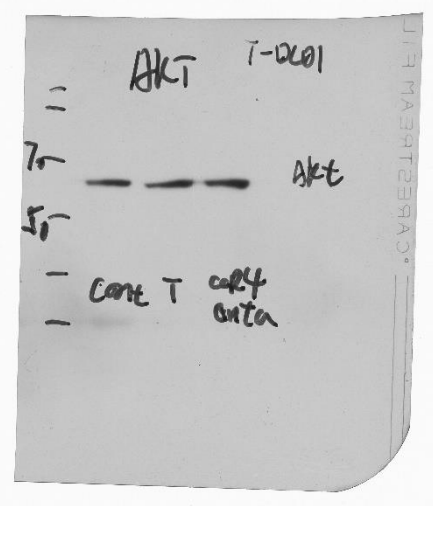

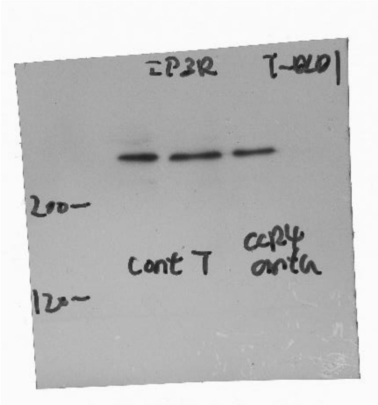

DLD1-AKT

DLD1-IP3R

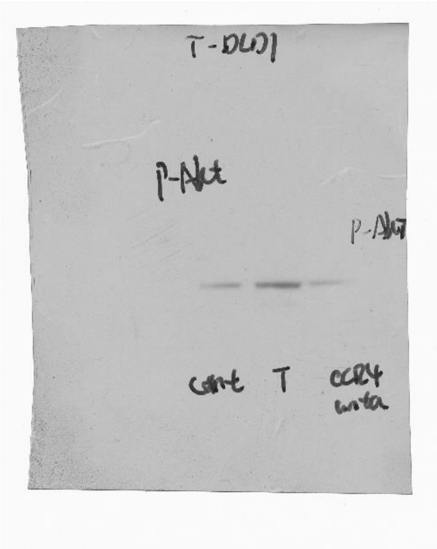

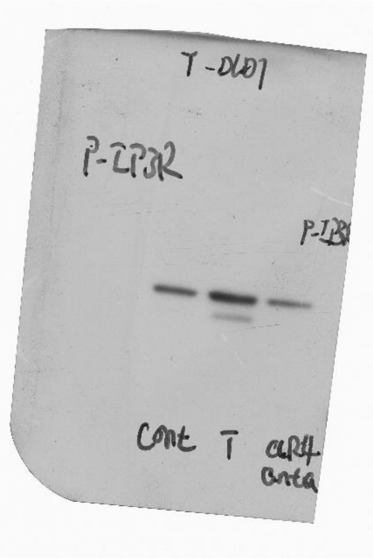

DLD1-p-AKT DLD1-p-IP3R

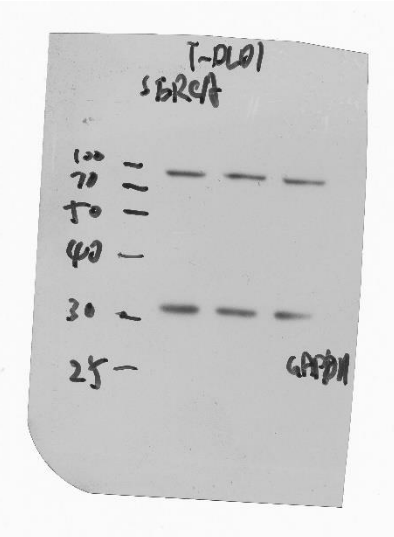

DLD1-SERCA-GAPDH

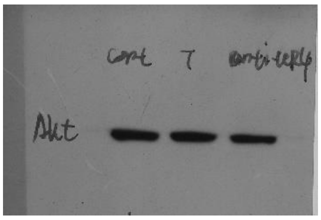

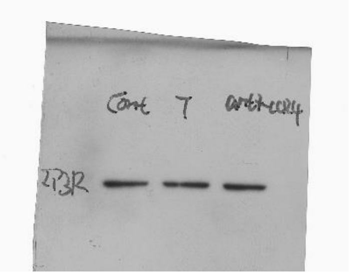

SW480-AKT SW480-IP3R

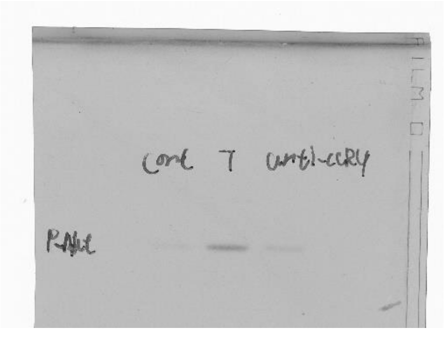

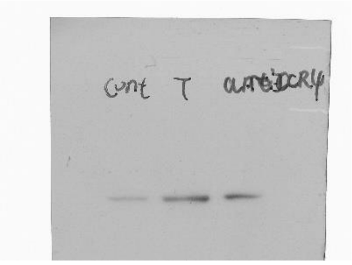

SW480-p-AKT

SW480-p-IP3R

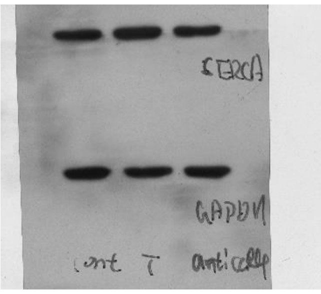

SW480-SERCA-GAPDH

Fig 5C

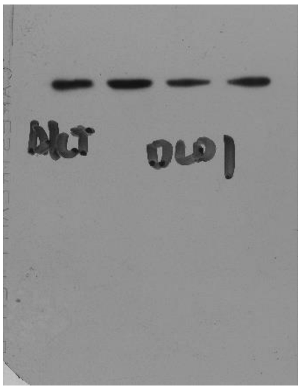

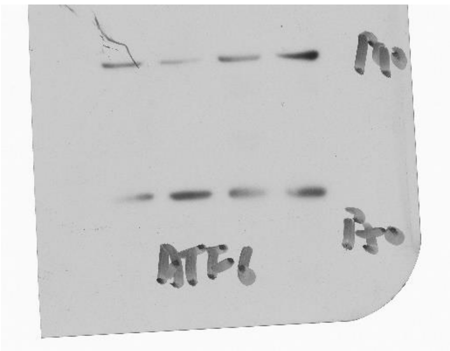

DLD1-AKT

DLD1-ATF6

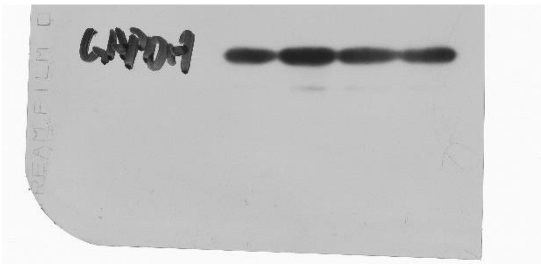

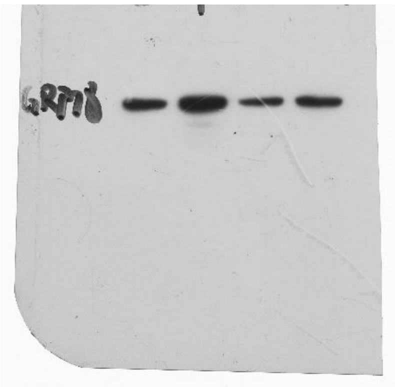

DLD1-GAPDH

DLD1-GRP78

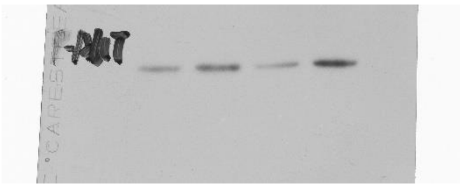

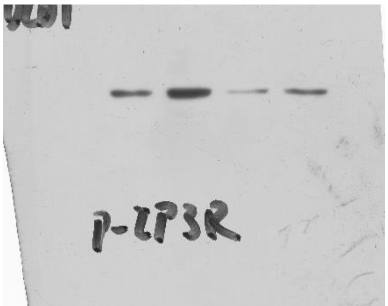

DLD1-p-AKT DLD1-p-IP3R

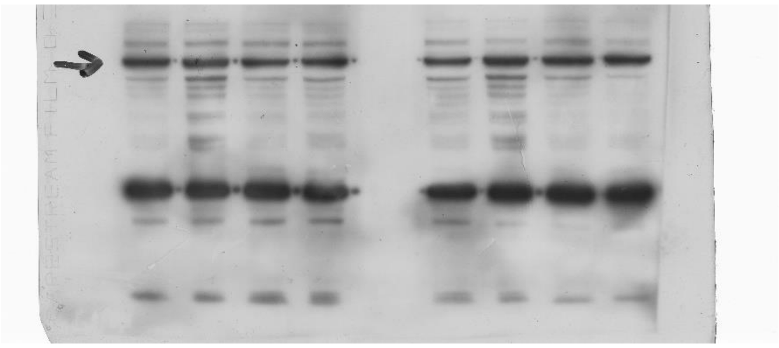

IP3R

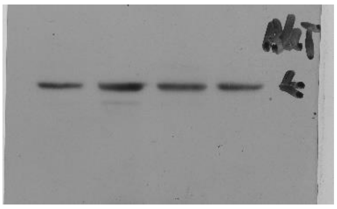

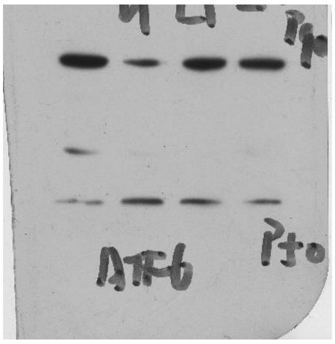

SW480-AKT

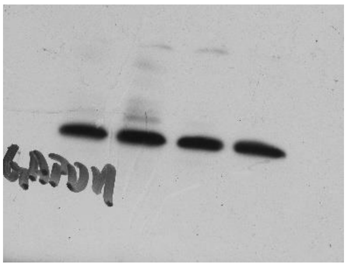

SW480-ATF6

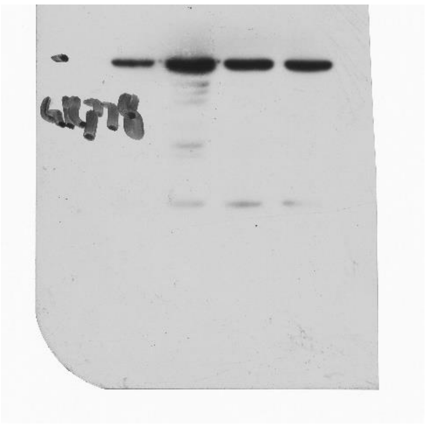

SW480-GAPDH

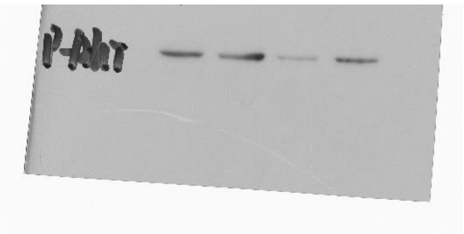

SW480-GRP78

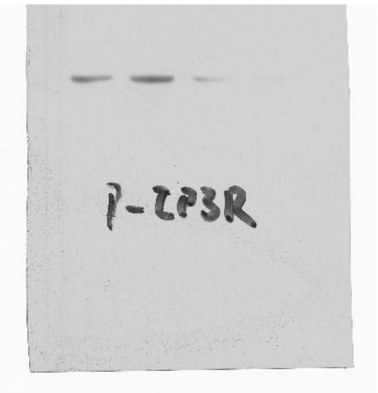

SW480-p-AKT SW480-p-IP3R

Fig 6C

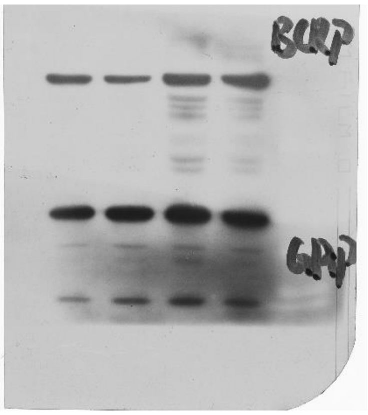

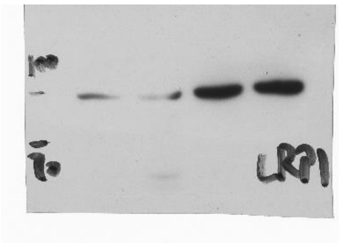

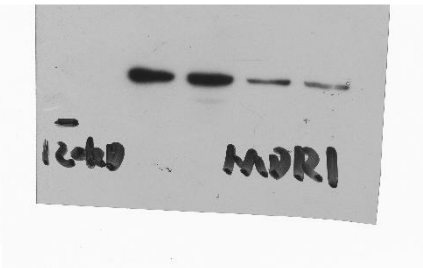

BCRP-GAPDH LRP1 MDR1

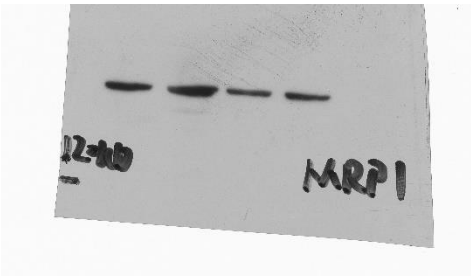

MRP1

Fig 6D

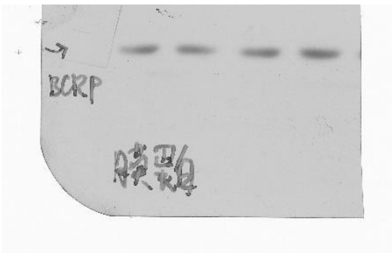

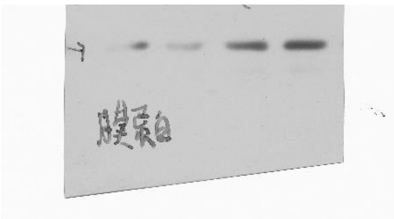

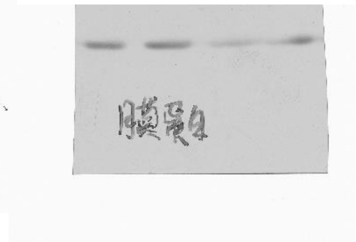

BCRP LRP MDR1

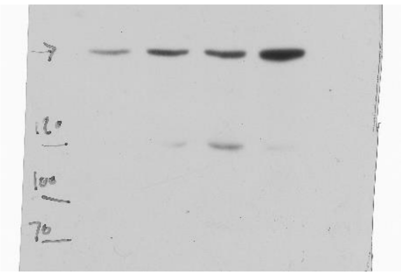

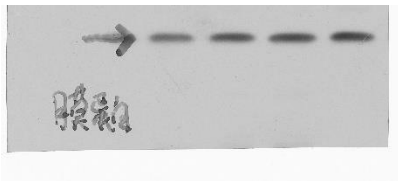

MRP1

Na-K-ATPase

Fig 6E

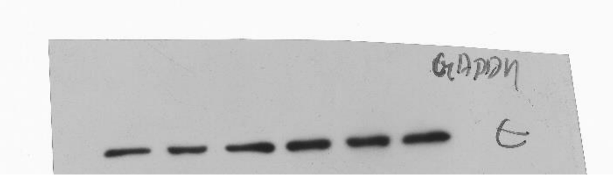

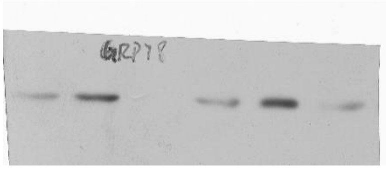

GAPDH

GRP78

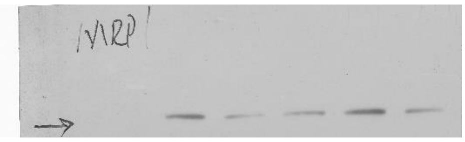

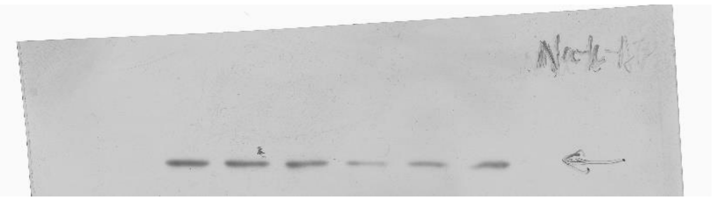

MRP1

Na-K-ATPase

Fig 6J

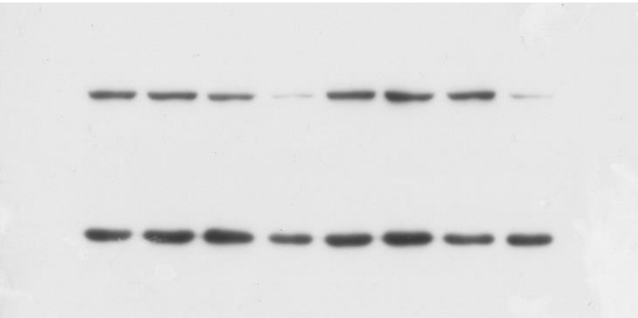

Fig 7A

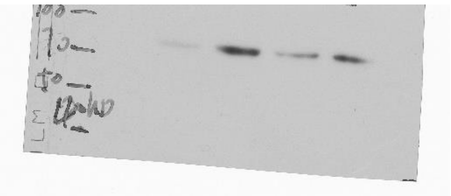

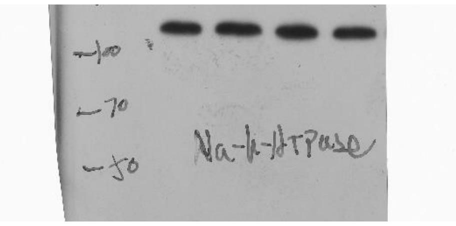

GRP78

Na-K-ATPase

Fig 7C

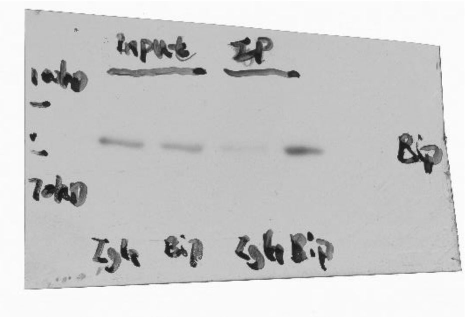

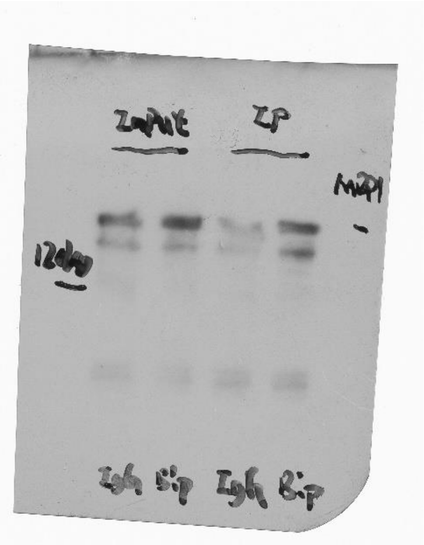

GRP78 MRP1

Fig 7D

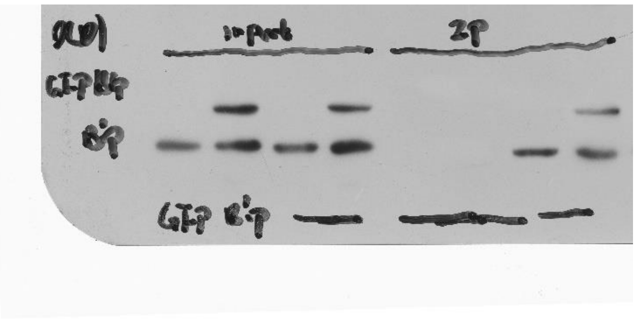

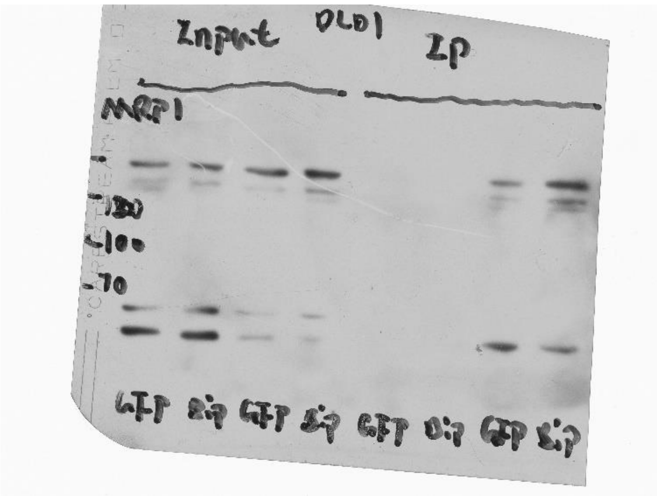

GRP78 MRP1

Fig 7F

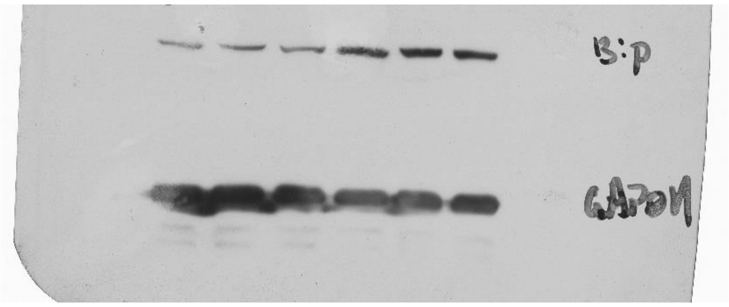

H-GRP78-GAPDH

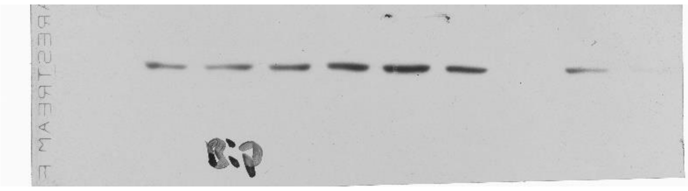

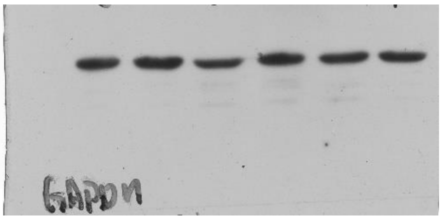

T-GRP78

T-GAPDH

Fig 7G

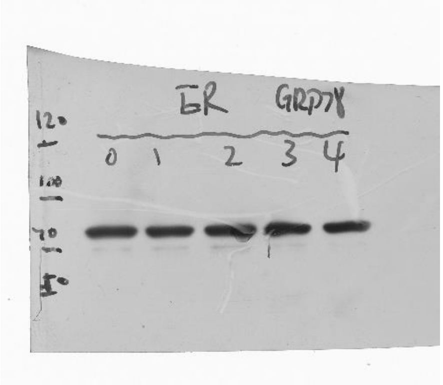

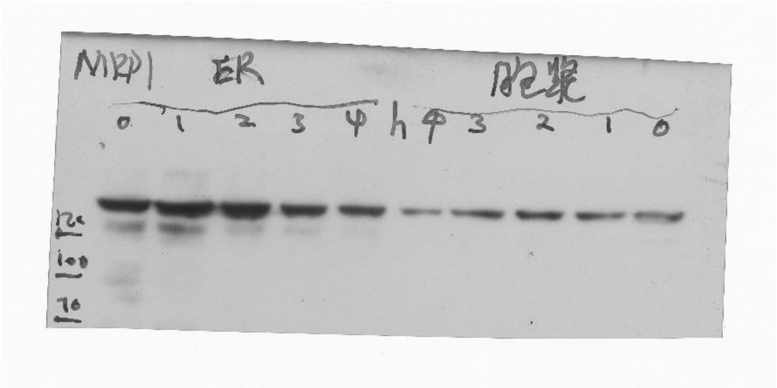

ER-GRP78

ER-plasm-MRP1

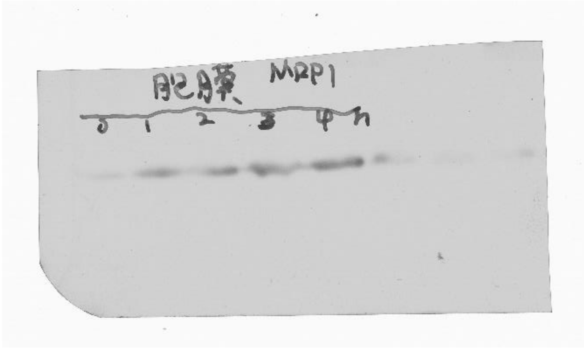

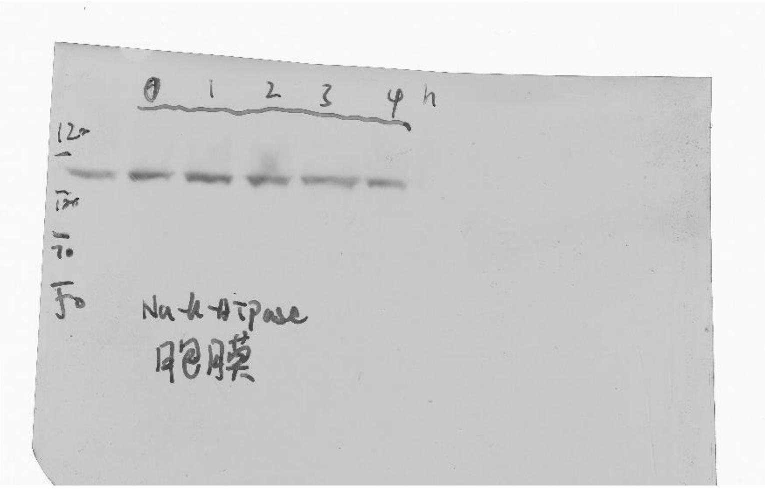

Memb-MRP1

memb-Na-K-ATPase

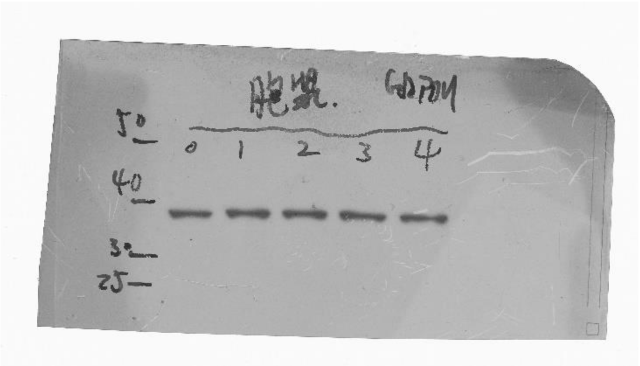

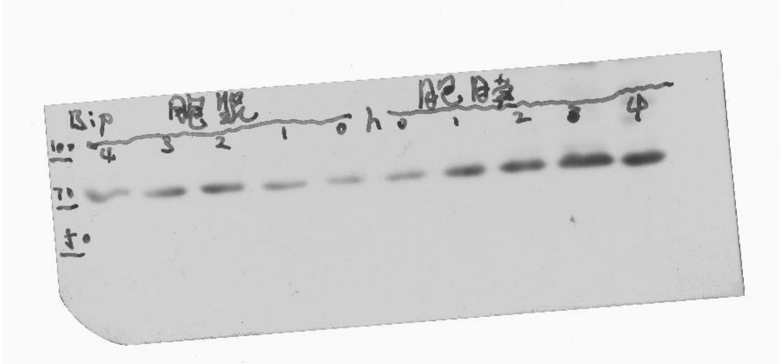

Plasm-GAPDH

plasm-memb-GRP78

Fig 7H

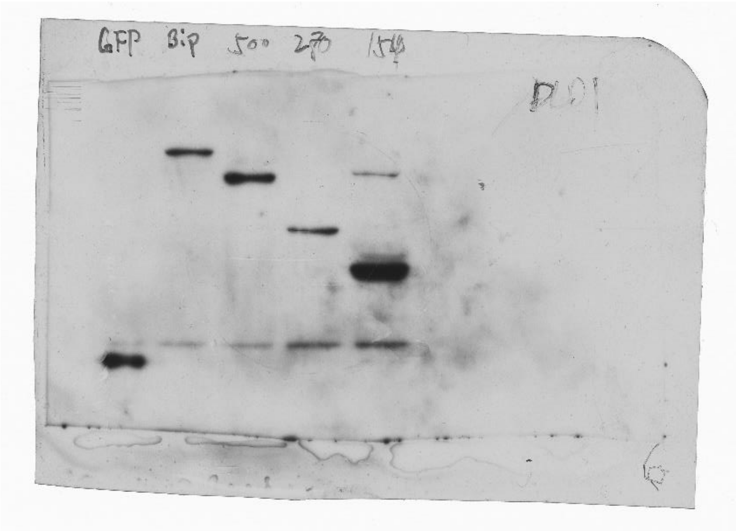

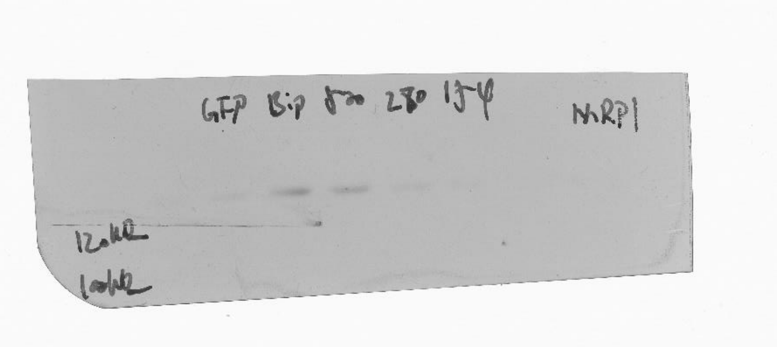

DLD1-GRP78

DLD1-MRP1

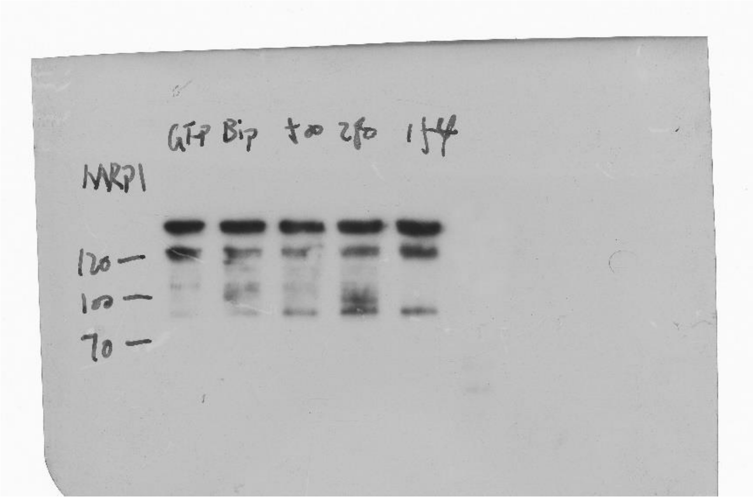

DLD1-input-MRP1

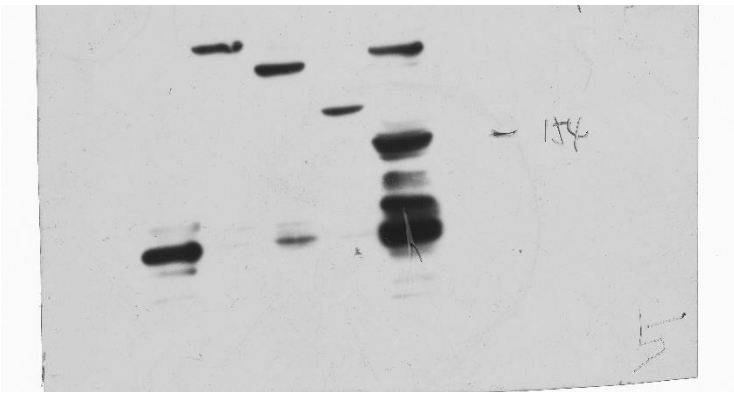

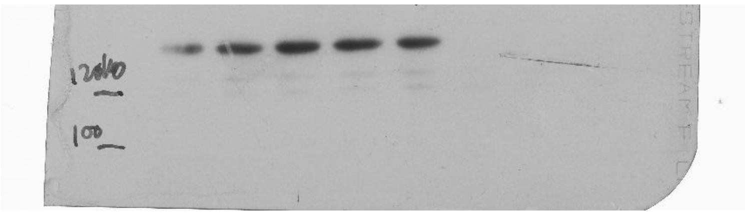

SW480-GRP78

SW480-input-MRP1

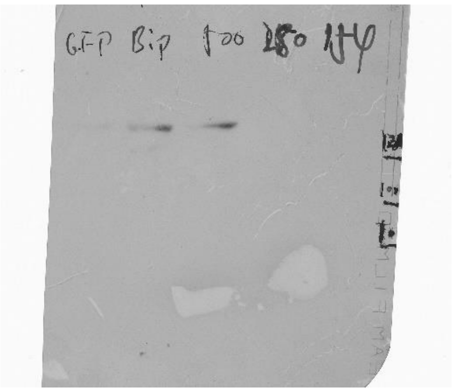

SW480-MRP1

Fig 7I

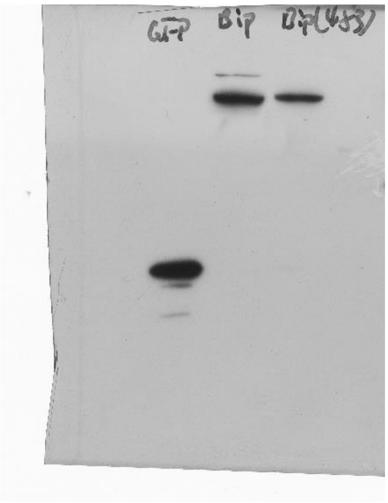

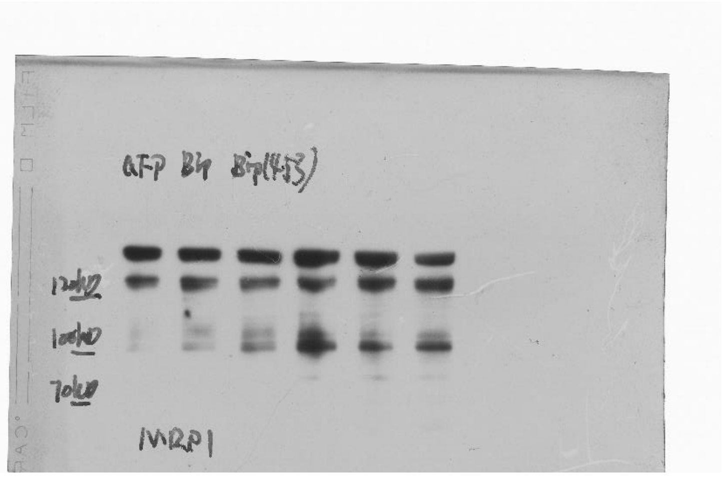

GRP78

input-MRP1

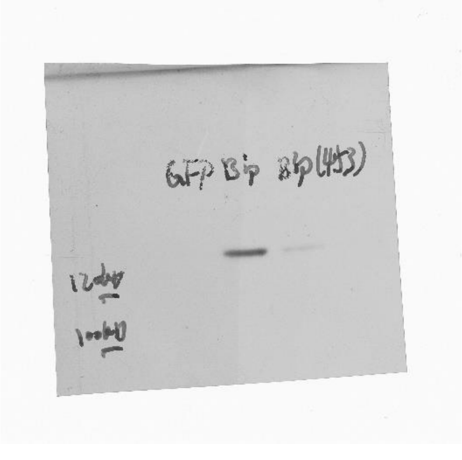

MRP1
