# Supplementary material for: Tumor-associated macrophages confer colorectal cancer 5-fluorouracil resistance by promoting MRP1 membrane translocation via an intercellular CXCL17/CXCL22–CCR4–ATF6–GRP78 axis
Source: Cell Death Dis. 2023 Sep 1;14(9):582. doi: 10.1038/s41419-023-06108-0 (PMC10474093; doi:10.1038/s41419-023-06108-0)
Supplement: Supplementary file 2 — Supplementary Figures and legends [file 41419_2023_6108_MOESM2_ESM.docx]

**Supplementary Information**

**M2 Macrophages Confer 5-Fluorouracil Resistance via Activation of CCR4 ATF6-GRP78 axis-mediated MRP1 Membrane Translocation in Colorectal Cancer**

**Supplementary Figures**

Figure S1. The identification of macrophage polarization derived from THP-1 and hPBMC.

Figure S2. The GO enrichment analysis of upregulated genes.

Figure S3. The GO enrichment analysis of downregulated genes.

Figure S4. Detection of CCL17 and CCL22 in macrophage-conditioned medium and CCR4 on CRC cell surface.

Figure S5. The validation of the key role of MRP1 in TAMs promoting 5-FU resistance of CRC.

Figure S6. The validation of MRP1 and GRP78 interaction.

Figure S7. The schematic of molding and drug delivery of *in vivo* mice experiment.

**Supplementary Tables**

Supplemental Table 1. The list of primers used in this manuscript

Supplemental Table 2. The list of genes and terms involved.

Supplemental Table 3. shRNA and siRNA sequences used in this study

**
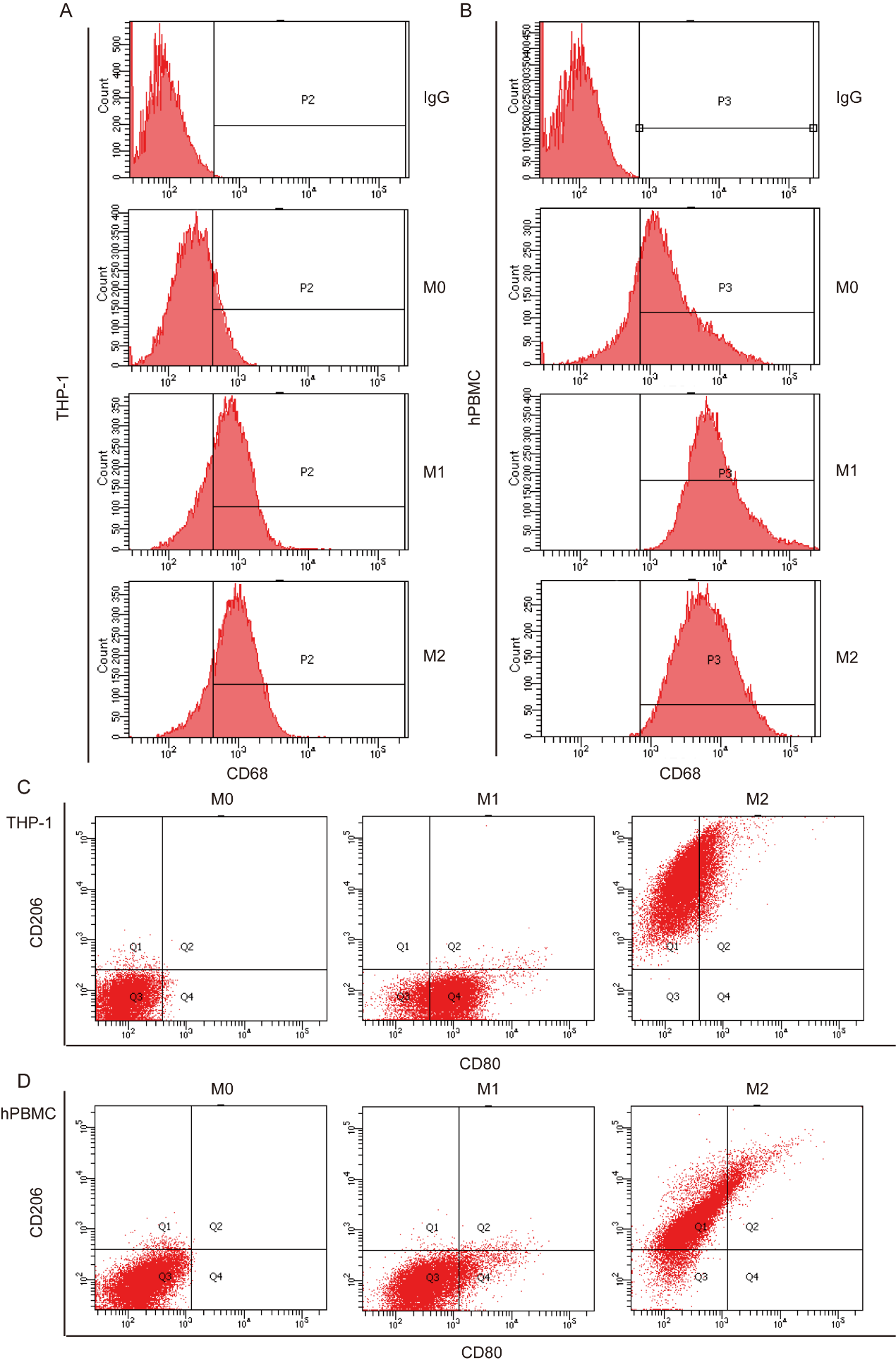
**

**Figure S1. The identification of macrophage polarization derived from THP-1 and hPBMC. (A)** CD68 was detected in THP-1-derived M0, M1, and M2 macrophages by flow cytometry. **(B)** CD68 was detected in hPBMC-derived M0, M1, and M2 macrophages by flow cytometry. **(C)** CD80 and CD206 were detected in THP-1-derived M0, M1, and M2 macrophages by flow cytometry. **(D)** CD80 and CD206 were detected in hPBMC-derived M0, M1, and M2 macrophages by flow cytometry.


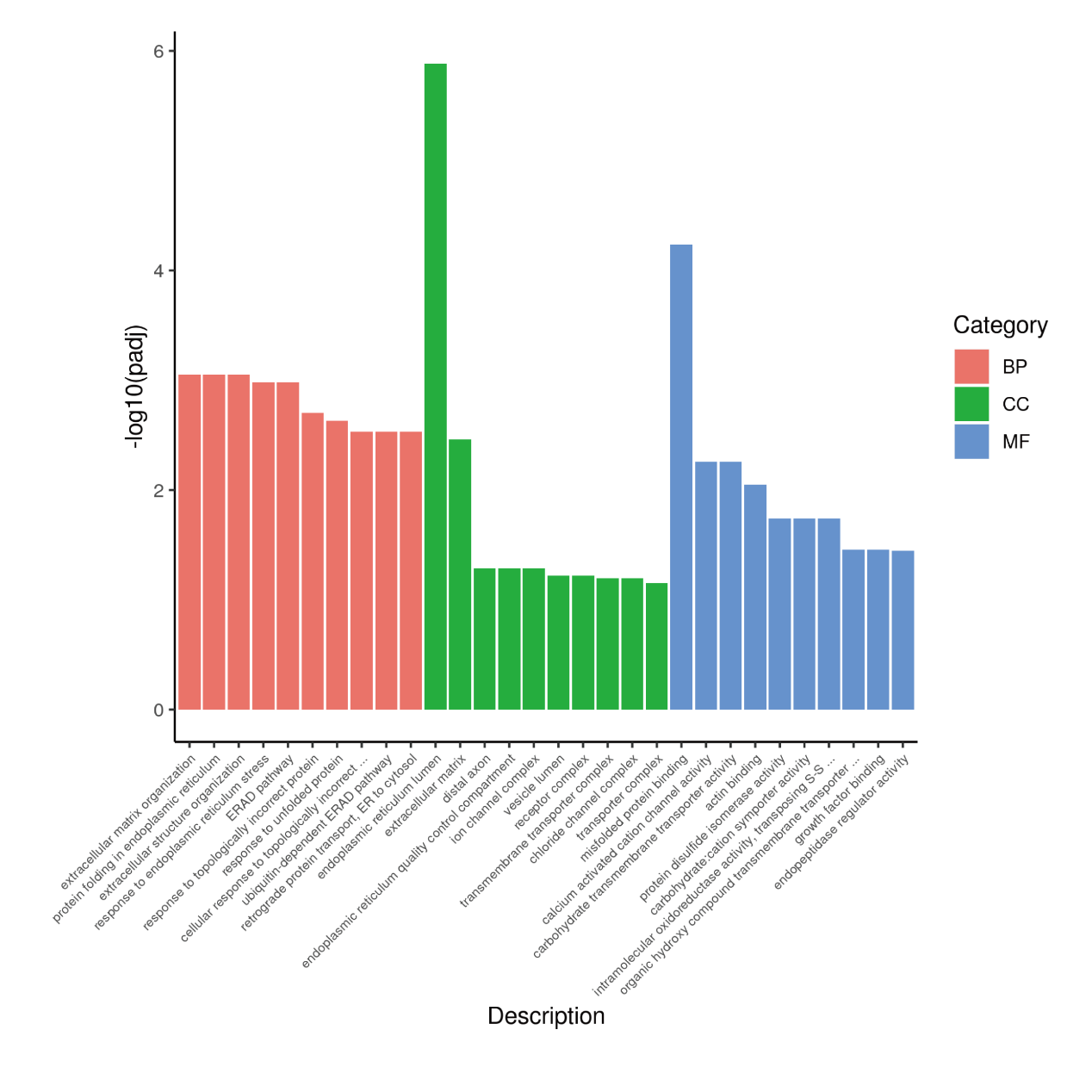


**Figure S2. The GO enrichment analysis of upregulated genes.** GO analysis identified a total of 30 terms, which containing 10 terms related to cellular components, 10 terms for biological processes, and 10 terms for molecular functions.


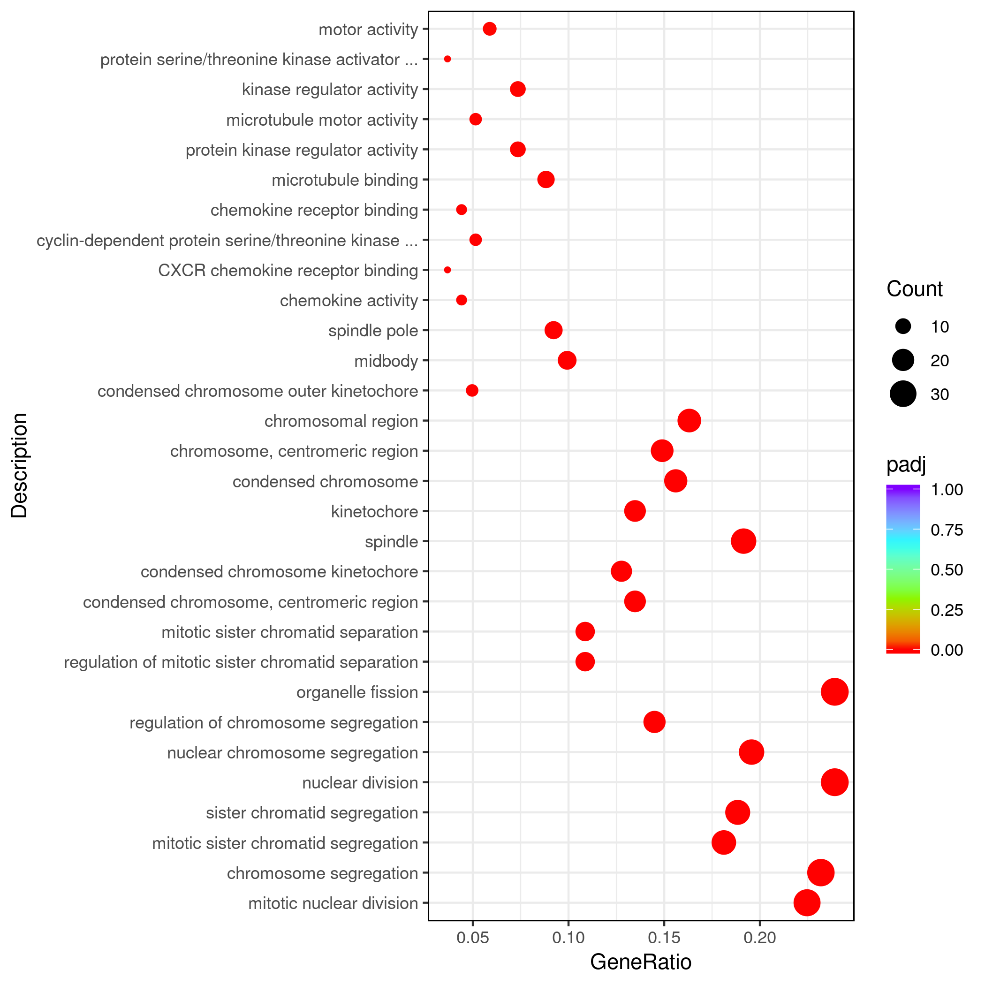


**Figure S3. The GO enrichment analysis of the 198 downregulated genes.** GO analysis identified a total of 30 terms.


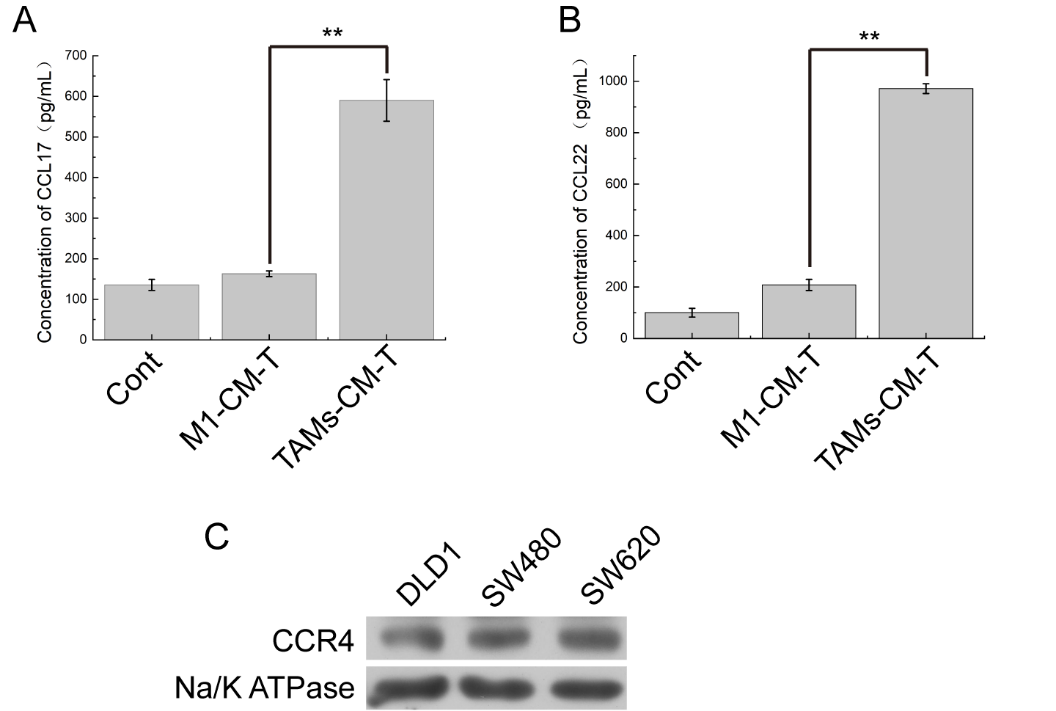


**Figure S4. Detection of CCL17 and CCL22 in macrophage-conditioned medium and CCR4 on CRC cell surface. (A)** ELISA detection of CCL17 in M1-CM-T and TAMs-CM-T, ***p* < 0.01. **(B)** ELISA detection of CCL22 in M1-CM-T and TAMs-CM-T, ***p* < 0.01. **(C)** Cell membrane proteins were extracted from DLD1, SW480, and SW620 cells, and the CCR4 was detected by Western blot.


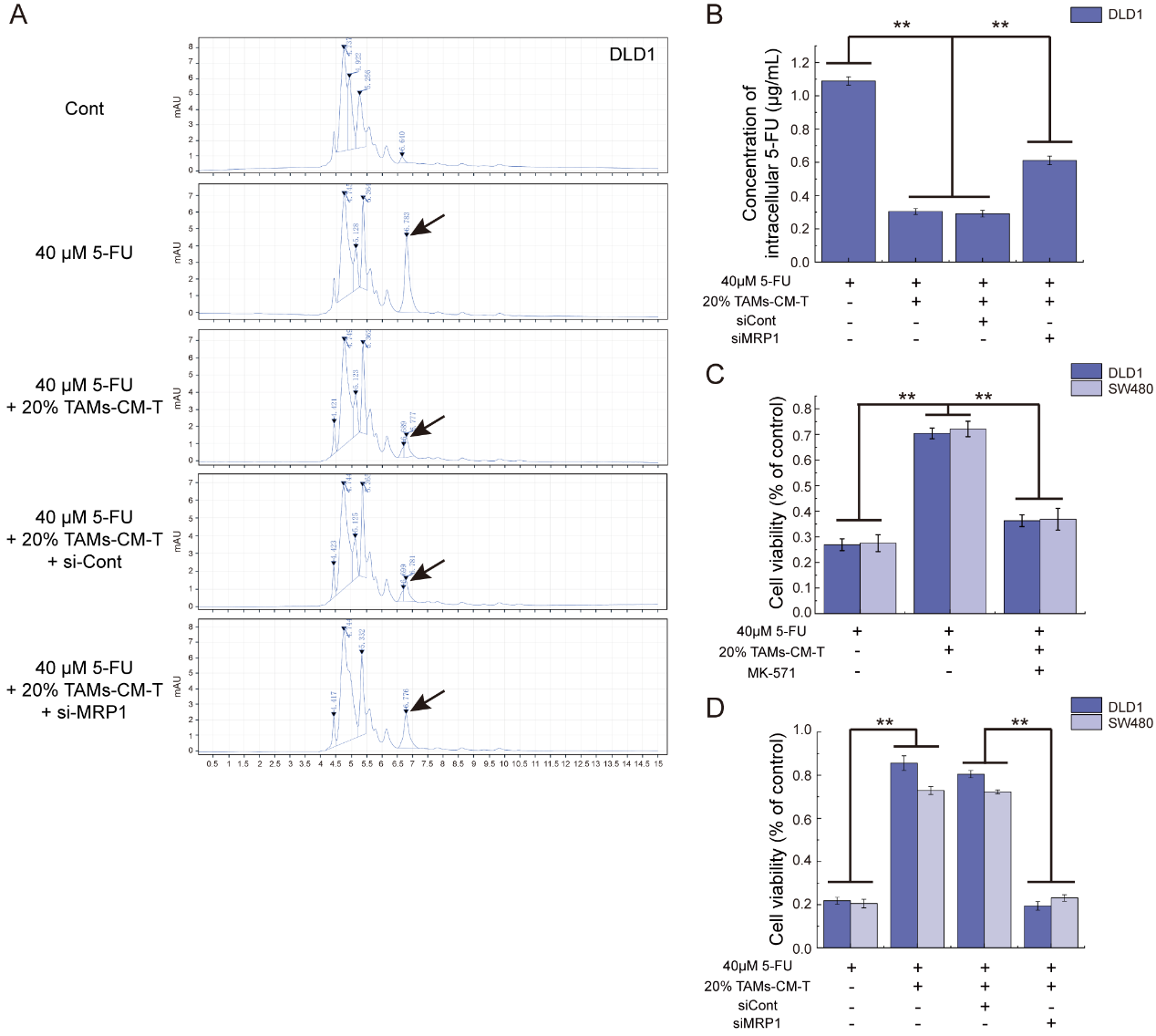


**Figure S5. The validation of the key role of MRP1 in TAMs promoting 5-FU resistance of CRC. (A)** After knocking down MRP1 by siRNA, 20% TAMs-CM-T was applied to evaluate its effects on the 5-FU efflux of DLD1 by HPLC. **(B)** The bar graph represented the concentration of 5-FU in DLD1 cells that were shown in A, ***p* < 0.01. **(C)** After inhibiting MRP1 by MK-571, the viability of cells was detected in DLD1 and SW480 cells which were treated with 20% TAMs-CM-T, ***p* < 0.01. **(D)** After knockdown MRP1 by siRNA, the viability of cells was detected in DLD1 and SW480 cells which were treated with 20% TAMs-CM-T, ***p* < 0.01.

**
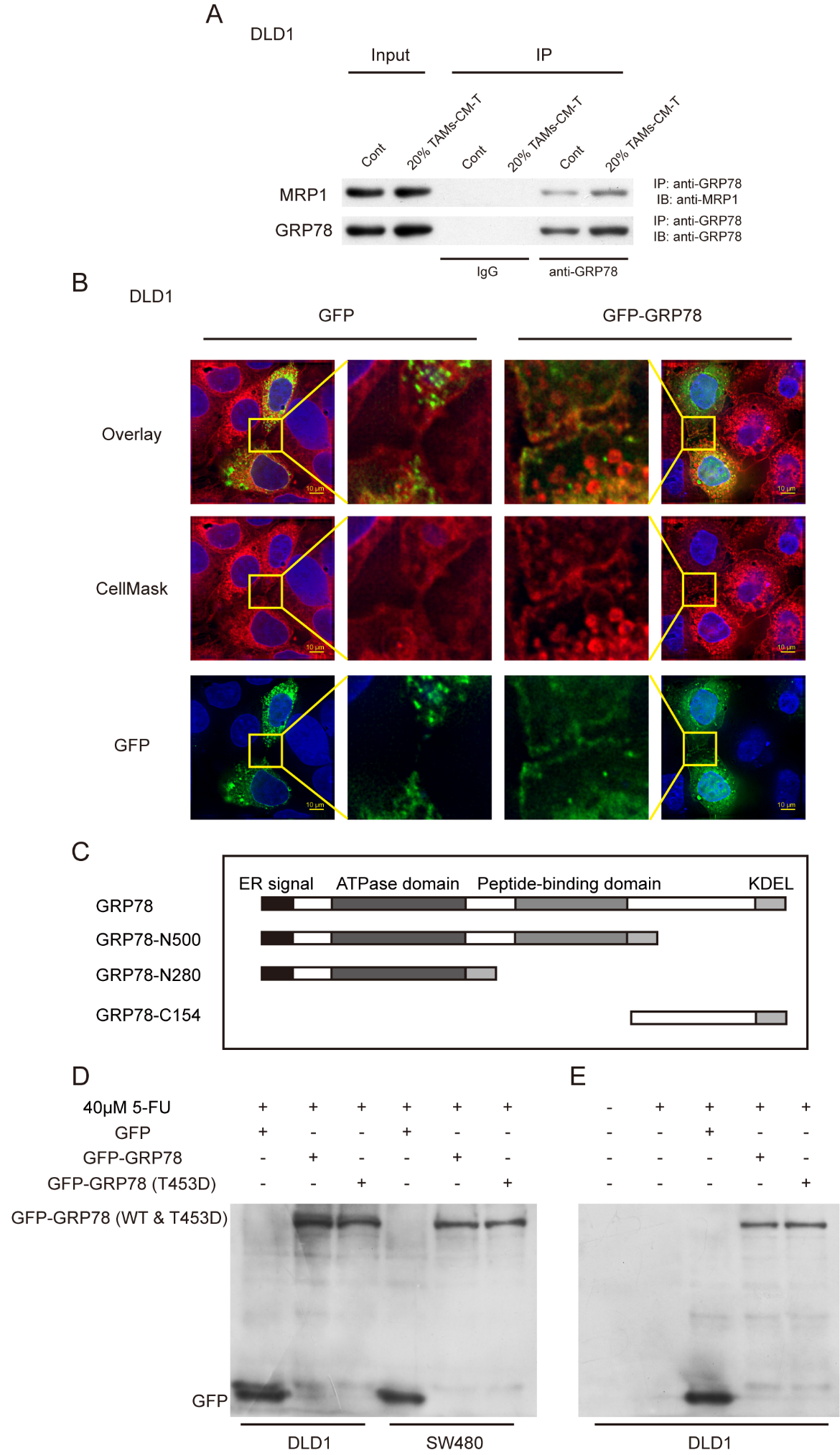
**

**Figure S6. The validation of MRP1 and GRP78 interaction. (A)** The interaction of GRP78 and MRP1 was detected by Co-IP in 20%-TAMs-CM-T-treated DLD1 cells. **(B)** With GFP-GRP78 overexpressed in DLD1 cells, the location of GFP-GRP78 was detected by immunofluorescence stain, GFP-GRP78 (green), and CellMask (cell membrane dye, red). **(C)** GRP78 consists of 654 amino acids, GRP78-N500 included 1-500 amino acids, GRP78-N280 included 1-280 amino acids, GRP78-C154 included 501-654 amino acids. **(D and E)** GRP78 (WT) and GRP78 (T453D) were overexpressed in DLD1 or SW480 cells, and the expression of the exogenous gene was detected by Western blot.


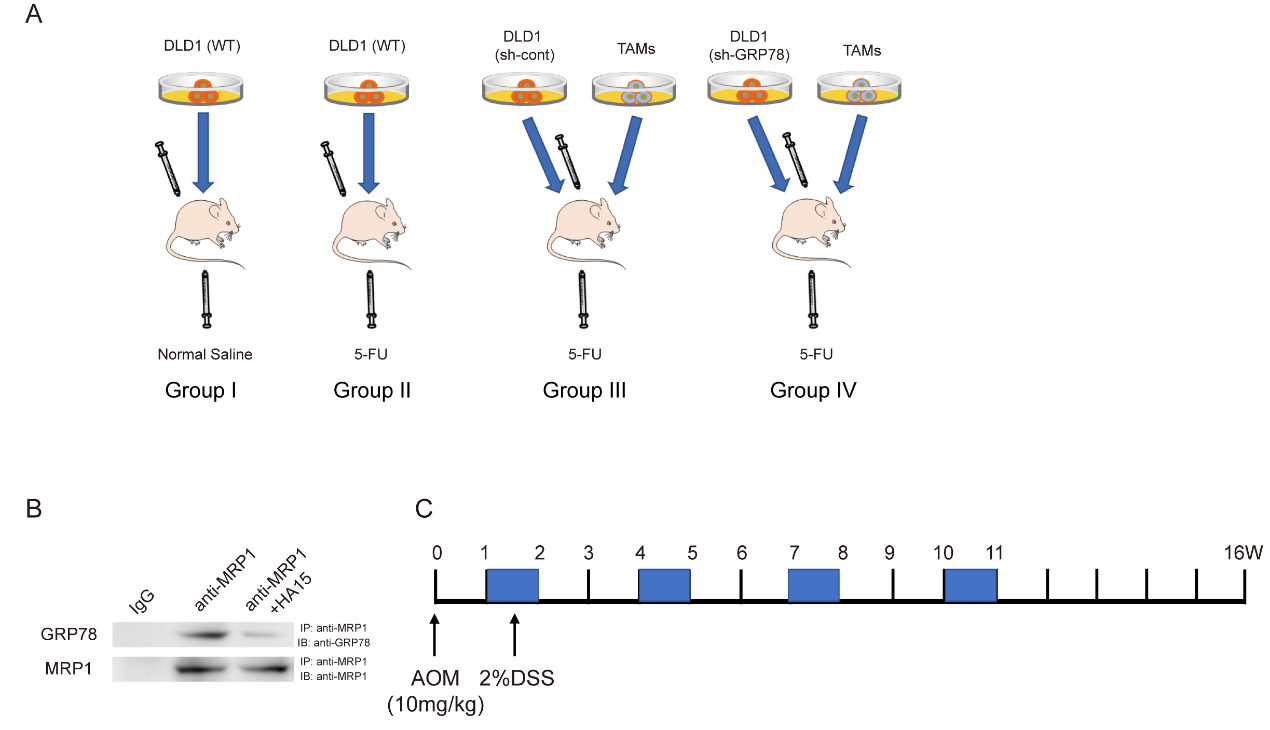


**Figure S7. The schematic of molding and drug delivery of *in vivo* mice experiment. (A)** the schematic of xenograft tumor assay. **(B)** With HA15 presence, the interaction of GRP78 and MRP1 was detected by Co-IP. **(C)** The schedule of AOM/DSS mouse colon tumor model

**Supplemental Table 1. The list of primers used in this manuscript**

| *GRP78* | 5′-CTGTGCAGCAGGACATCAAGTTC-3′ |
| --- | --- |
|  | 5′-TGTTTGCCCACCTCCAATATCA-3′ |
| *CCL17* | 5′-AGGGACCTGCACACAGAGAC-3′ |
|  | 5′-CTCGAGCTGCGTGGATGTGC-3′ |
| *CCL22* | 5′-ATGGCTCGCCTACAGACTGCACTC-3′ |
|  | 5′-CACGGCAGCAGACGCTGTCTTCCA-3′ |
| *LRP* | 5′-AGCCAGCTATGCACCAACAC-3′ |
|  | 5′-CCTTGCAGGAGCGGTTATC-3′ |
| *BCRP* | 5′-ACGAACGGATTAACAGGGTCA-3′ |
|  | 5′-CTCCAGACACACCACGGAT-3′ |
| *MRP-1* | 5′-CTCTATCTCTCCCGACATGACC-3′ |
|  | 5′-AGCAGACGATCCACAGCAAAA-3′ |
| *MDR* | 5′-GGGAGCTTAACACCCGACTTA-3′ |
|  | 5′-GCCAAAATCACAAGGGTTAGCTT-3′ |
| *GAPDH* | 5′-GCACCGTCAAGGCTGAGAAC-3′ |
|  | 5′-TGGTGAAGACGCCAGTGGA-3′ |

**Supplemental Table 2. The list of gene and terms involved.**

| **Gene name** | **log2 Fold Change** | **Number of terms involved** | **Terms name** |
| --- | --- | --- | --- |
| *GRP78* | 2.147447374 | 9 | response to endoplasmic reticulum stress; response to topologically incorrect protein; response to unfolded protein; cellular response to topologically incorrect protein; ERAD pathway; ubiquitin-dependent ERAD pathway; protein folding in endoplasmic reticulum; misfolded protein binding; endoplasmic reticulum lumen |
| *HSP90B1* | 1.303247236 | 9 | response to endoplasmic reticulum stress; response to topologically incorrect protein; response to unfolded protein; cellular response to topologically incorrect protein; ERAD pathway; ubiquitin-dependent ERAD pathway; protein folding in endoplasmic reticulum; endoplasmic reticulum lumen; retrograde protein transport, ER to cytosol |
| *EDEM1* | 1.087803167 | 9 | response to endoplasmic reticulum stress; response to topologically incorrect protein; response to unfolded protein; cellular response to topologically incorrect protein; ERAD pathway; ubiquitin-dependent ERAD pathway; misfolded protein binding; endoplasmic reticulum quality control compartment; retrograde protein transport, ER to cytosol |
| *DERL2* | 1.005191523 | 9 | response to endoplasmic reticulum stress; response to topologically incorrect protein; response to unfolded protein; cellular response to topologically incorrect protein; ERAD pathway; ubiquitin-dependent ERAD pathway; misfolded protein binding; endoplasmic reticulum quality control compartment; retrograde protein transport, ER to cytosol |
| *DNAJB9* | 1.578296846 | 8 | response to endoplasmic reticulum stress; response to topologically incorrect protein; response to unfolded protein; cellular response to topologically incorrect protein; ERAD pathway; ubiquitin-dependent ERAD pathway; misfolded protein binding; endoplasmic reticulum lumen |
| *HERPUD1* | 1.570422794 | 7 | response to endoplasmic reticulum stress; response to topologically incorrect protein; response to unfolded protein; cellular response to topologically incorrect protein; ERAD pathway; ubiquitin-dependent ERAD pathway; retrograde protein transport, ER to cytosol |
| *DNAJC3* | 1.556787286 | 7 | response to endoplasmic reticulum stress; response to topologically incorrect protein; response to unfolded protein; cellular response to topologically incorrect protein; protein folding in endoplasmic reticulum; misfolded protein binding; endoplasmic reticulum lumen |
| *CALR* | 1.383336796 | 7 | response to endoplasmic reticulum stress; response to topologically incorrect protein; response to unfolded protein; cellular response to topologically incorrect protein; protein folding in endoplasmic reticulum; endoplasmic reticulum lumen; endoplasmic reticulum quality control compartment |
| *SDF2L1* | 1.155148215 | 6 | response to endoplasmic reticulum stress; response to topologically incorrect protein; cellular response to topologically incorrect protein; ERAD pathway; misfolded protein binding; endoplasmic reticulum lumen |
| *STT3B* | 1.174768073 | 5 | response to endoplasmic reticulum stress; response to topologically incorrect protein; response to unfolded protein; ERAD pathway; ubiquitin-dependent ERAD pathway; |
| *SEL1L* | 1.14158357 | 5 | response to endoplasmic reticulum stress; ERAD pathway; ubiquitin-dependent ERAD pathway; endoplasmic reticulum quality control compartment; retrograde protein transport, ER to cytosol |
| *DNAJB11* | 1.102162751 | 5 | response to endoplasmic reticulum stress; response to topologically incorrect protein; response to unfolded protein; cellular response to topologically incorrect protein; endoplasmic reticulum lumen |
| *HSPA6* | 2.757808248 | 4 | response to topologically incorrect protein; response to unfolded protein; cellular response to topologically incorrect protein; misfolded protein binding |
| *MANF* | 1.596655503 | 4 | response to endoplasmic reticulum stress; response to topologically incorrect protein; response to unfolded protein; endoplasmic reticulum lumen |
| *DDIT3* | 1.502550626 | 4 | response to endoplasmic reticulum stress; response to topologically incorrect protein; response to unfolded protein; cellular response to topologically incorrect protein; |
| *BHLHA15* | 1.218382837 | 4 | response to endoplasmic reticulum stress; response to topologically incorrect protein; response to unfolded protein; cellular response to topologically incorrect protein; |
| *BRSK2* | 1.37623393 | 3 | response to endoplasmic reticulum stress; ERAD pathway; retrograde protein transport, ER to cytosol |
| *FBXO2* | 1.119259561 | 3 | response to endoplasmic reticulum stress; ERAD pathway; ubiquitin-dependent ERAD pathway; |
| *PDIA4* | 1.525346565 | 2 | response to endoplasmic reticulum stress; endoplasmic reticulum lumen |
| *COL13A1* | 2.875166491 | 1 | endoplasmic reticulum lumen |
| *COL15A1* | 2.788046368 | 1 | endoplasmic reticulum lumen |
| *COL6A3* | 2.577323189 | 1 | endoplasmic reticulum lumen |
| *ANO1* | 2.284206754 | 1 | calcium activated cation channel activity |
| *TRPM5* | 2.232573211 | 1 | calcium activated cation channel activity |
| *LGALS1* | 1.920500572 | 1 | endoplasmic reticulum lumen |
| *CERCAM* | 1.539947318 | 1 | endoplasmic reticulum lumen |
| *PTPRN2* | 1.4428494 | 1 | endoplasmic reticulum lumen |
| *MATN3* | 1.371307953 | 1 | endoplasmic reticulum lumen |
| *SELENOM* | 1.347327704 | 1 | endoplasmic reticulum lumen |
| *FSTL3* | 1.328854789 | 1 | endoplasmic reticulum lumen |
| *KCNN4* | 1.247373149 | 1 | calcium activated cation channel activity |
| *ERO1B* | 1.229771543 | 1 | protein folding in endoplasmic reticulum |
| *SERPINA1* | 1.219450599 | 1 | endoplasmic reticulum lumen |
| *COL6A1* | 1.216775884 | 1 | endoplasmic reticulum lumen |
| *TMEM63C* | 1.087291597 | 1 | calcium activated cation channel activity |
| *COLGALT2* | 1.08658237 | 1 | endoplasmic reticulum lumen |
| *RASGRF1* | 1.082219583 | 1 | response to endoplasmic reticulum stress |
| *MSLN* | 1.018746502 | 1 | endoplasmic reticulum lumen |
| *CATSPER1* | 1.009143384 | 1 | calcium activated cation channel activity |

**Supplemental Table 3. shRNA and siRNA sequences used in this study**

| *sh-GRP78-1* | 5’-CCGGAGATTCAGCAACTGGTTAAAGCTCGAGC TTTAACCAGTTGCTGAATCTTTTTTG-3’ |
| --- | --- |
| *sh-GRP78-2* | 5’-CCGGGAGCGCATTGATACTAGAAATCTCGAGA TTTCTAGTA TCAATGCGCTCTTTTTG-3’ |
| *sh-Control* | 5’-CCGGCCTAAGGTTAAGTCGCCCTCGCTCGAGC GAGGGCGACTTAACCTTAGGTTTTTG-3’ |
| *si-ATF6* | 5′-GGCAGGACUACGAAGUGAUGA-3′ |
| *Si-MRP1* | 5′-GAGGCUUUGAUCGUCAAGUTT-3′ |
| *si-Control* | 5′-AUCGACCUUACCGCUAACAUGC-3′ |
